# Deep Learning for the Industrial Internet of Things (IIoT): A Comprehensive Survey of Techniques, Implementation Frameworks, Potential Applications, and Future Directions

**DOI:** 10.3390/s21227518

**Published:** 2021-11-12

**Authors:** Shahid Latif, Maha Driss, Wadii Boulila, Zil e Huma, Sajjad Shaukat Jamal, Zeba Idrees, Jawad Ahmad

**Affiliations:** 1 School of Information Science and Engineering, Fudan University, Shanghai 200433, China; lshahid19@fudan.edu.cn (S.L.); izeba17@fudan.edu.cn (Z.I.); 2 Security Engineering Lab, Prince Sultan University, Riyadh 12435, Saudi Arabia; mdriss@psu.edu.sa; 3 RIADI Laboratory, National School of Computer Science, University of Manouba, Manouba 2010, Tunisia; wboulila@psu.edu.sa; 4 Robotics and Internet of Things Lab, Prince Sultan University, Riyadh 12435, Saudi Arabia; 5 Department of Electrical Engineering, Institute of Space Technology, Islamabad 44000, Pakistan; zilehuma@mail.ist.edu.pk; 6 Department of Mathematics, College of Science, King Khalid University, Abha 61413, Saudi Arabia; shussain@kku.edu.sa; 7 School of Computing, Edinburgh Napier University, Edinburgh EH10 5DT, UK

**Keywords:** artificial intelligence, deep learning, internet of things, industrial internet of things, smart industry

## Abstract

The Industrial Internet of Things (IIoT) refers to the use of smart sensors, actuators, fast communication protocols, and efficient cybersecurity mechanisms to improve industrial processes and applications. In large industrial networks, smart devices generate large amounts of data, and thus IIoT frameworks require intelligent, robust techniques for big data analysis. Artificial intelligence (AI) and deep learning (DL) techniques produce promising results in IIoT networks due to their intelligent learning and processing capabilities. This survey article assesses the potential of DL in IIoT applications and presents a brief architecture of IIoT with key enabling technologies. Several well-known DL algorithms are then discussed along with their theoretical backgrounds and several software and hardware frameworks for DL implementations. Potential deployments of DL techniques in IIoT applications are briefly discussed. Finally, this survey highlights significant challenges and future directions for future research endeavors.

## 1. Introduction

**Figure 1:**
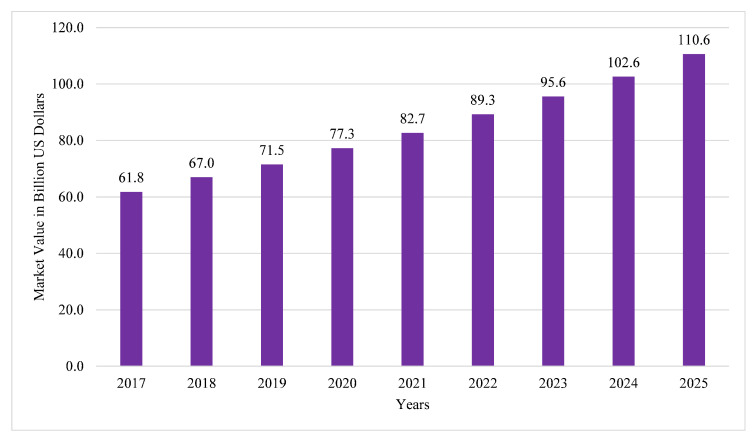
The worldwide market size of the IIoT from 2017 to 2025 [5].

The Internet of Things (IoT) describes a ubiquitous connection between common objects and the Internet. IoT functions by deploying thousands of smart devices in living or industrial environments. These devices collect the information from surrounding environments, perform desired processing activities on the data acquired and transmit that processed data through secure, reliable communication channels. Recent advancements in software, hardware, and communication systems have significantly improved human lifestyles in terms of time, energy, and cost savings [1,2]. The term Industrial Internet of Things (IIoT) refers to the use of conventional IoT concepts in industrial applications. The IIoT improves manufacturing procedures by enabling sustainable and efficient technologies in an industrial environment [3,4]. The IIoT market is currently undergoing rapid growth as well as increasing adaptations in the digital transformations of many industries. Strong alliances and concurrences of interests between IIoT stakeholders and emerging applications has attracted major companies worldwide to invest in this emerging market. According to the latest Statistica report, the size of the IIoT market is expected to increase up to $110.6B USD by 2025 [5]. A comparative analysis of the IIoT market and its increase in size from 2017 to projections for 2025 is presented in Figure 1.

**Figure 2:**
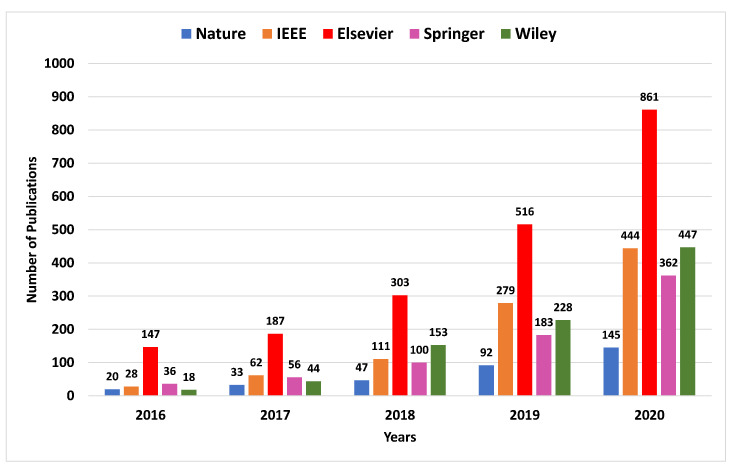
A comparison of publication records for DL-based IoT/IIoT applications.

In the fourth industrial revolution, a rapid increase in the number of interconnected devices in the IIoT systems has been recorded. In the IIoT, the performance of smart applications is heavily associated with the intelligent processing of big data. Therefore, IIoT networks require intelligent information processing frameworks for big data analysis. In this context, artificial intelligence (AI), and particularly DL, techniques can be very useful as a means of producing effective, usable results from big data in an IIoT system [4,6,7]. For instance, DL techniques enable the system’s capacities to learn from experience. The efficiency and effectiveness of a DL solution depend upon the characteristics and nature of the data in question as well as the performance of the learning algorithm [8]. The selection of a suitable DL algorithm for a specific application can be a challenging task. Therefore, it is essential to understand the workflow of various DL algorithms and their deployment in real-world applications such as smart homes, smart cities, cybersecurity services, smart healthcare, smart transportation, sustainable agriculture, business enterprises, and many others [9]. The significance of DL in IoT/IIoT applications can be determined by analyzing the emerging and cutting-edge research in this area. The bar chart in Figure 2 presents a comparative analysis of relevant publications records by the top publishers from 2016 to 2020.

### 1.1. Existing Surveys on DL for IoT and IIoT Applications

In recent literature, multiple DL-based solutions for IoT and IIoT applications have been proposed by both academia and industry. In the following section, some of the latest survey articles are briefly described to demonstrate the depth and scope of existing literature.

Mohammadi et al. [10] have presented a detailed overview of DL techniques and their importance to big data analysis for IoT applications. They also discuss some of the most-used DL algorithms as well as their backgrounds and architectures. They also summarized the deployment of DL techniques in fog and cloud-based IoT environments. They conclude this work by presenting some potential challenges and directions for future research. Meanwhile Ma et al. [11] presented a comprehensive examination of how leveraging DL techniques in IoT applications could be possible. The main goal of this survey is to present the applicability of DL techniques to empower multiple applications in fields including smart healthcare, robotics, home monitoring, traffic control, autonomous driving, and manufacturing. In another survey, Sengupta et al. [12] explored the potential of DL approaches for multiple applications including perspective and predictive analysis, financial time series forecasting, power systems, medical image processing, etc.

Ambika et al. [13] presented a comprehensive survey about ML and DL techniques along with their architectures and impact on IIoT. This survey also discussed some use cases of ML and DL approaches in the IoT and IIoT domains. Saleem et al. [14] then presented a concise discussion on the potential benefits of DL algorithms for IoT applications. This survey formulated DL-based human activity recognition systems and presented a brief comparison of different DL models before discussing advanced research options in the realm of DL-driven IoT applications. As these authors pointed out, with the development of the IoT, several real-time apps collect data about people and their surroundings using IoT sensors and input it into DL models to improve an application’s intelligence and capabilities, allowing it to provide better suggestions and services. In this regard, Deepan and Sudha [15] presented a self-contained review of several DL models for real-time IoT applications. Additionally, the authors provided a better understanding of DL models and their efficiency by adding different DL tools and frameworks. In one of the most recent and best survey articles, Khalil et al. [6] discussed the key potential of DL techniques in IIoT applications. Here the authors reviewed different DL algorithms and their use in different industries. They outlined numerous use cases of DL for IIoT, including smart agriculture, smart manufacturing, smart metering, etc. Moreover, several key challenges and future research directions were discussed.

### 1.2. Limitations of Existing Surveys

A detailed comparison of the aforementioned surveys is presented in Table 1, demonstrating how researchers have done well in providing a perspective on DL-based IoT and IIoT applications. However, the existing literature has a few notable limitations. First, most of these researchers discussed only the theoretical aspects of DL for IIoT, but not real-time deployment. Second, there is a need for reference architecture of IIoT with key enabling technologies. Third, most of the surveys discussed the mathematical and theoretical background of DL algorithms, but the implementation frameworks and hardware platforms for real-time deployments are not considered or discussed adequately. Finally, these studies used only a limited description of the use cases of DL algorithms in the IIoT sector. Therefore, to overcome these various limitations, we present a comprehensive study on DL technologies for IIoT applications.

### 1.3. Major Contributions

This study presents a comprehensive deliberation on recent advancements in the integration of DL technologies with IIoT. The major contributions of this survey are as follows.

1. Discussing the great potential of DL schemes for IIoT.
2. Providing a detailed reference architecture of the IIoT with key enabling technologies.
3. Presenting a comprehensive survey on the working principle of well-known DL algorithms, their implementation frameworks, and the relevant hardware platforms.
4. Covering the real-world applications of DL techniques in IIoT systems.
5. Suggesting some potential future research directions.

The remainder of this article is organized as follows. Section 2 describes the detailed reference architecture of the IIoT system. Section 3 briefly discusses the potential of several DL schemes for IIoT and elaborates the working principle of each algorithm. Some well-known software frameworks are discussed in relation to the implementation of DL algorithms in Section 4. Section 5 presents some suitable hardware platforms for the real-time implementation of DL schemes for the IIoT. Section 6 comprises some real-world cases of DL application for IIoT and Section 7 summarizes the challenges faced by DL-based IIoT and presents some potential future research directions. Finally, a brief conclusion is presented in Section 8.

## 2. Reference Architecture of the Industrial Internet of Things (IIoT)

**Figure 3:**
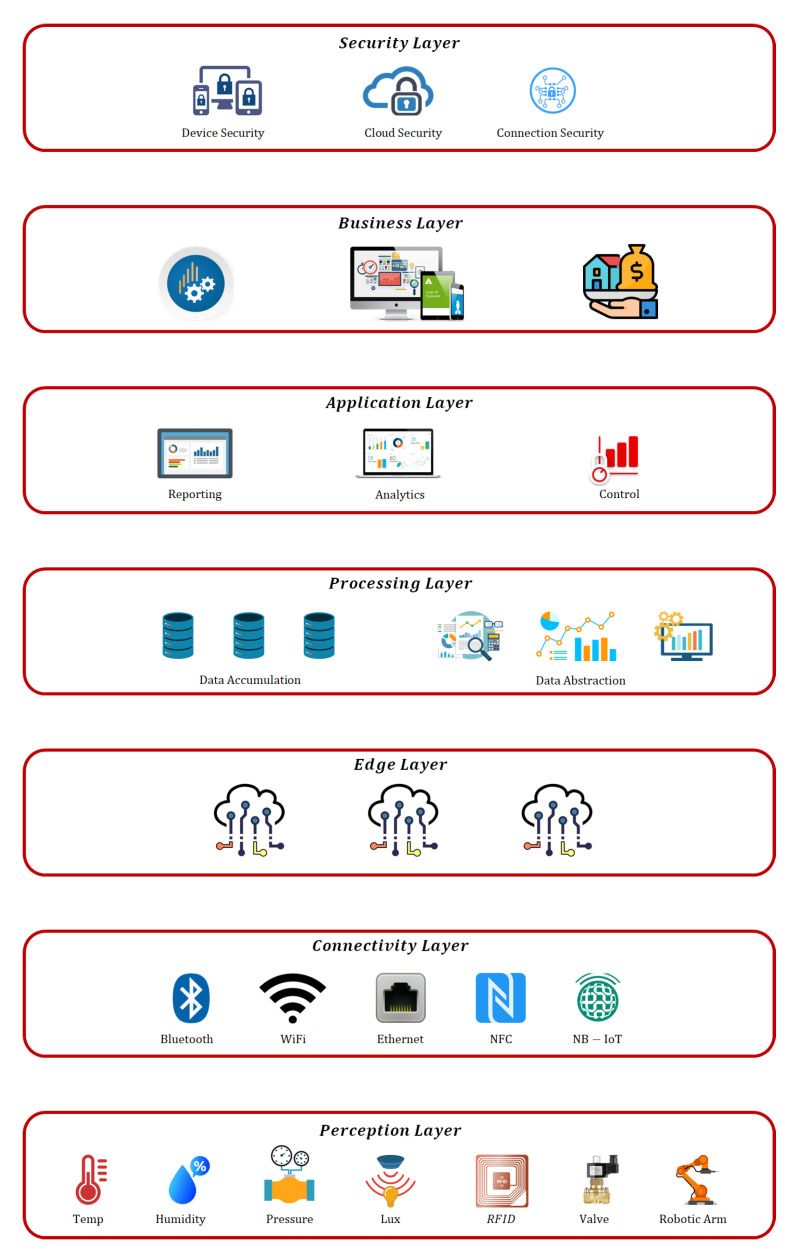
Reference architecture of the Industrial Internet of Things.

Over the past decade, IIoT has brought about a great revolution in modern technologies by providing seamless connectivity among devices. In this Internet-enabled era, our mobile applications, devices (smartwatches, tablets/iPads, laptops, etc.), and many more are sending valuable information continuously to IoT networks. These IoT/IIoT systems are built upon specific architectures that include different layers and elements with particular functionalities [16,17]. For instance, a detailed 7-layer architecture for the IIoT is presented in Figure 3.

### 2.1. Perception Layer

This layer, which is also known as a “physical” layer, collects environmental data and performs the necessary preprocessing. This layer transforms the acquired analog data into digital to make it best compatible with other layers of the system [18]. The main components of this layer are sensors and actuators.

- Sensors: These are small devices that can detect changes in the surrounding environment and extract useful information from the data acquired. Usually, sensors are considered to be resource-constrained devices that have little processing and computational power combined with limited memory resources. However, modern sensors have great capacity to gather environmental signals with a higher level of accuracy. The most commonly used sensors across multiple industries are used to measure temperature, humidity, air pressure, weight, acceleration, position, and many others.
- Actuators: These are usually electromechanical devices that convert electrical signals into physical actions. In an industrial environment, linear and rotary are the two general types of actuators most often used. Linear actuators transform electrical signals into linear motions, which are useful in position adjustment applications. Meanwhile, rotary actuators transform electrical energy into rotational energy. These are usually used for the position control of devices such as conveyor belts.

### 2.2. Connectivity Layer

This layer connects the perception layer and edge layer using advanced communication technologies [19]. Two methods may be adopted for communication in this layer. In the first method, direct communication is performed using a TCP or UDP/IP stack. In another method, smart gateways provide a communication link between the local area network (LAN) and the wide-area network (WAN). Several advanced communication technologies and protocols are used in this layer, as described in the following.

- WiFi: This is the most versatile and commonly used scheme across communication technologies. WiFi modems are very suitable for both personal and official uses, delivering smooth communications among LAN and WAN.
- Ethernet: This is an older technology used for connecting devices in a LAN or WAN, which enables them to communicate with each other via a specific communication protocol. Ethernet also enables network communication between different network cables, such as copper to optical fiber and vice versa.
- Bluetooth: This wireless protocol is widely used for information exchange over short distances by creating personal area networks.
- NFC: Near Field Communications (NFC) is a wireless technology that enables secure communication between smart devices at short distances. The communication range of NFC is usually considered to be about 10 cm.
- LPWAN: Low-Power Wide-Area Network (LPWAN) describes a class of radio technologies used for long-distance communication. The top LPWAN technologies are LoRa, Sigfox, and Nwave. As compared to other wireless technologies such as Bluetooth and Wi-Fi, LPWANs are usually used to send smaller amounts of information over longer distances.
- ZigBee: This is a product from the Zigbee alliance that is designed especially for sensor networks on the IEEE 802.15.4 standard. The mostly used data communication protocols for this communication standard are ISA-100.11.a and WirelessHART. These protocols define Media Access Control (MAC) and physical layers to handle several devices at low-data rates.
- LTE-M: Long Term Evolution for Machine is a leading LPWA network technology for IoT applications. It is used for interconnecting objects such as IoT sensors and actuators, or other industrial devices via radio modules.
- NB-IoT: This is a standards-based low-power wide-area (LPWA) technology that enables a wide variety of smart devices and services. NB-IoT improves the power consumption, spectrum efficiency, and system capacity of smart devices.

### 2.3. Edge Layer

In the early stages, latency often becomes a major challenge to the growth of an IoT network. Edge computing is considered to be a potential solution to this issue, as it accelerates the growth of IoT networks. This layer facilitates the system’s ability to process and analyze data closer to the source. Edge computing technology has now become a new standard for advanced 5G mobile networks that enable broader system connectivity at lower latency. The performance of IoT networks is significantly improved by performing all processes at the edge [20].

### 2.4. Processing Layer

This layer collects, stores, and analyses information from the edge layer [21]. These operations are all performed by IoT systems and include two main stages.

- Data Accumulation: Real-time information is acquired through an API and further stored to meet the demands of non-real-time applications. This stage serves as a transient link between query-based data consumption and event-based data generation. This stage also determines the relevance of the data acquired to the stated business requirements.
- Data Abstraction: Once data accumulation and preparation have been completed, consumer applications may use it to produce insights. Several phases are involved in the end-to-end process, including integrating data from multiple sources, reconciliation of formats, and data aggregation in a single place.

To enhance the compatibility of smart devices, these techniques are usually deployed together. The commonly used protocols for the processing layers are described in the following.

- Transmission Control Protocol (TCP): It offers host-to-host communication, breaking large sets of data into individual packets and resending and reassembling packets as needed.
- User Datagram Protocol (UDP): Process-to-process communication is enabled using this protocol, which operates on top of IP. Over TCP, UDP offers faster data transfer speeds, making it the protocol of choice for mission-critical applications.
- Internet Protocol (IP): Many IoT protocols use IPv4, while more recent executions use IPv6. This recent update to IP routes traffic across the Internet and identifies and locates devices on the network.

### 2.5. Application Layer

In this layer, software analyses the data to provide promising solutions to critical business problems. Thousands of IIoT applications vary in design complexity and functions, as demonstrated by various versions of this layer. Each one uses various technologies and operating systems (OS). Some prominent applications include device monitoring through software control, business intelligence (BI) services, analytic solutions through AI techniques, and mobile applications for simple interactions. In recent trends, the application layer can be built at the top of IoT/IIoT frameworks that provide software-based architectures with ready-made instruments for data visualization, data mining, and data analytics [22]. Some widely used protocols for the applications layer are discussed in the following.

- Advanced Message Queuing Protocol (AMQP): It allows messaging middleware to communicate with one another. It enables a variety of systems and applications to communicate with one another, resulting in standardized communications on a large scale.
- Constrained Application Protocol (CoAP): A constrained-bandwidth and constrained-network protocol designed for machine-to-machine communication between devices with limited capacity. CoAP is a document-transfer protocol that operates on the User Datagram Protocol (UDP).
- Data Distribution Service (DDS): A flexible peer-to-peer communication protocol capable of running small devices as well as linking high-performance networks. DDS simplifies deployment, boosts dependability, and minimizes complexity.
- Message Queue Telemetry Transport (MQTT): A messaging protocol developed for low-bandwidth communications to faraway places and mostly used for lightweight machine-to-machine communication. MQTT employs a publisher-subscriber pattern and is suited for tiny devices with limited bandwidth and battery life.

### 2.6. Business Layer

The IoT-acquired data are considered useful if they are applicable for business planning and strategy. Every business has specific goal-oriented tasks that must be accomplished by extracting useful information from the data gathered. Business enterprises also use past and present data to plan future goals. Modern firms frequently adopt intelligent data analysis techniques to enhance their decision-making capabilities [23]. Today, software and business intelligence techniques have gained great attention from industries as a means of improving their performance and profitability.

### 2.7. Security Layer

Security has become one of the essential requirements of an IIoT architecture, due to the rapid increase of modern challenges. Hacking, denial of service, malicious software, and data breaches are the main challenges of the current IIoT infrastructures [24]. This layer performs three main functions as, which can be described as follows.

- Device Security: This is the beginning point of security in the IIoT framework. Many manufactures and companies integrate both software and hardware-based security schemes in IoT devices.
- Cloud Security: Cloud storage is replacing the traditional data storage servers in modern IoT infrastructures, so in turn new security mechanisms are also adopted to secure that cloud. Cloud security includes encryption schemes and intrusion detection systems, etc., as means of preventing cyberattacks and other malicious activities.
- Connection Security: In an IIoT network, the data must be encrypted before transmission via any communication channel. In this context, different messaging protocols, such as MQTT, DDS, and AMQP, may be implemented to secure valuable information. In modern trends, the use of TSL cryptographic protocol is recommended for communication in industrial applications.

## 3. Deep Learning for the IIoT

The use of smart manufacturing in the IIoT has several advantages. For one thing, it can be very helpful to make manufacturing and production processes intelligent [25]. New industrialists are adopting IIoT solutions to enhance the productivity and profitability of their industries. In IoT-enabled industries, the data collected from sensors and smart devices plays an important role to make the production process smarter [25]. Therefore, the deployment of intelligent data analysis techniques has now become an essential requirement of modern industries.

DL is considered to be one of the most powerful techniques in the domain of artificial intelligence (AI). The integration of DL methods in smart industries can upgrade the smart manufacturing process into a highly optimized environment by information processing through its multi-layer architecture. DL approaches are very helpful due to their inherited learning capabilities, underlying patterns identification, and smart decision-making. The biggest advantage of DL over conventional ML techniques is automatic feature learning. With this option, there is no need to implement a separate algorithm for feature learning [25].

The deployment of DL techniques can be very effective to perform the types of aforementioned analysis in smart industries. Some well-known DL-based techniques for the IIoT are discussed in the following section.

### 3.1. Deep Feedforward Neural Networks

**Figure 4:**
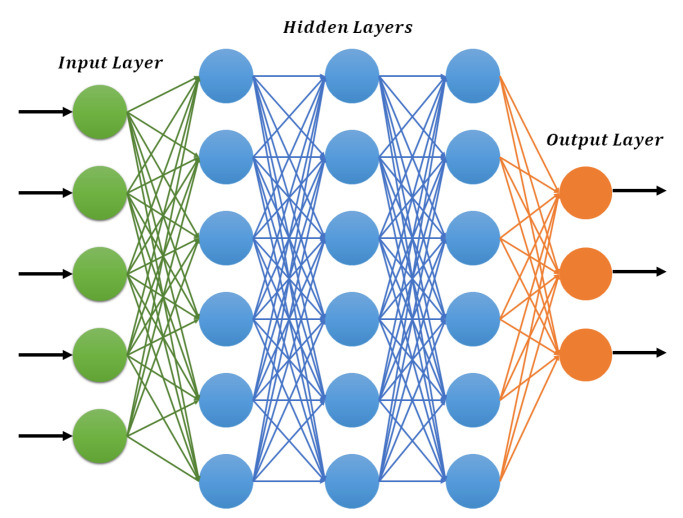
A general architecture of the deep feedforward neural network (DFNN).

This is the most fundamental type of deep neural network (DNN), in which the connections between nodes move forward. The general architecture of the deep forward neural network (DFNN) is presented in Figure 4. As compared to shallow networks, the multiple hidden layers in DNN can be very helpful to model complex nonlinear relations. This architecture is very popular in all fields of engineering because of its simplicity and robust training process [26].

The most commonly used gradient descent algorithm is preferred for the training of DFNNs. In this algorithm, the weights are first initialized randomly and then the error is minimized using gradient descent. The complete learning process involves multiple executions of forward and backward propagation consecutively [27]. In forward propagation, the input is processed towards the output layer through multiple hidden layers and the calculated output is compared with the desired output of the corresponding input. In the backward process, the rate of change of error is calculated concerning network parameters to adjust the weights. This process will continue until the desired output is achieved by the neural network.

Let $x_{i}$ as the input of neural network and $f_{i}$ as the activation function of layer *i*. The output of the layer *i* can be computed as

(1) $$x_{i+1}=f_{i}\left(w_{i}x_{i}+b_{i}\right)$$

Here, $x_{i+1}$ becomes the input for the next layer, $w_{i}$ and $b_{i}$ are the essential parameters that connect the layer *i* with the previous layer. These parameters are updated during the backward process as shown in the following.

(2) $$w_{new}=w-\eta \frac{\partial E}{\partial W}$$

(3) $$b_{new}=b-\eta \frac{\partial E}{\partial b}$$

Here, $w_{new}$ and $b_{new}$ are the updated parameters for *w* and *b*. *E* represents the cost function and η is the learning rate. The cost function of the DL model is decided according to the desired task such as classification, regression etc.

### 3.2. Restricted Boltzmann Machines (RBM)

**Figure 5:**
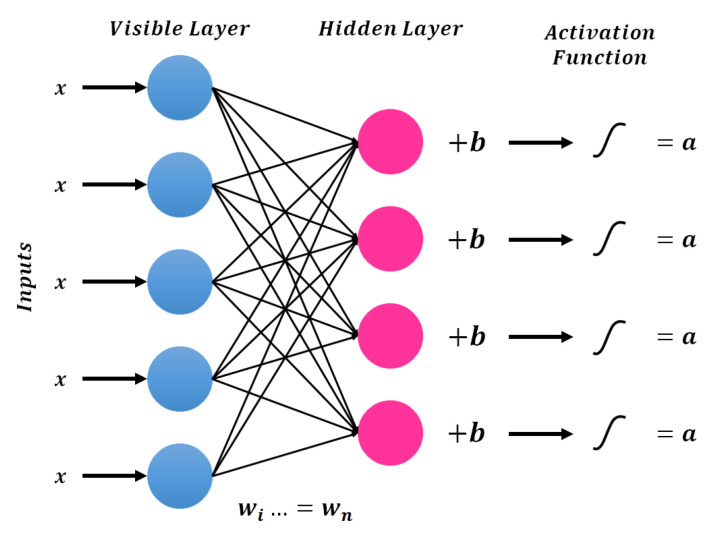
A general architecture of the Restricted Boltzmann Machines (RBM).

RBM can also be described as stochastic neural networks. This is one of the well-known and widely used DL approaches because of its capability to learn the input probability distribution in both supervised and unsupervised manners. Paul Smolensky introduced this technique in 1986 with the name Harmonium, and Hinton popularized it in 2002 with the development of new training algorithms [28]. Since then, RBM has been applied widely in a variety of tasks such as dimensionality reduction, representation learning, and prediction problems. Deep belief network training using RMB as a building block was a very famous application. Presently, RBMs are frequently used for collaborative filtering, due to the excellent performance of the Netflix dataset [29]. RBM is an extension of the Boltzmann Machine with a restriction in the intra-layer connection between units. It is an undirected graphical model that contains two layers, visible and hidden, that form a bipartite graph. In recent studies, several variations of RBMs have been introduced in terms of improvised learning algorithms [30]. These variants include conditional RBMs, temporal RBMs, convolutional RBMs, factored conditional RBMs, and Recurrent RBMs. To deal with the data characteristics, different types of nodes may be introduced, such as Gaussian, Bernoulli, etc. In RBM, each node is considered a computational unit that processes the information needed to make stochastic decisions. The general architecture of RBM is presented in Figure 5.

The joint probability distribution of a standard RMB can be described with the Gibbs distribution $p\left(v,h\right)=\frac{1}{z}e^{-E(v,h)}$

Here energy function E(v,h) can be described as

(4) $$E\left(v,h\right)=-\sum_{i=1}^{n}\sum_{j=1}^{m}w_{ij}h_{j}v_{i}-\sum_{j=1}^{m}b_{j}v_{i}-\sum_{i=1}^{n}c_{i}h_{i}$$

Here *m* and *n* represent the number of visible and hidden units. $h_{j}$ and $v_{j}$ are the states of the hidden unit *i* and visible unit *j* respectively. $b_{j}$ and $c_{j}$ describes real-valued biases corresponding to the jth and ith units, respectively. $w_{ij}$ are the weights that connect visible units with hidden units. *Z* indicates the normalization constant that ensures the sub-probability distributions equal to 1. Hinton proposed the Contrastive Divergence algorithm for the training of RBMs. This algorithm trains the RMB to maximize the training sample’s probability. Training stabilizes the model through minimization of its energy through updating the model’s parameters, which can be done using the following Equations (5)–(7).

(5) $$\Delta w_{ij}=\epsilon \left({\langle v_{i}h_{j}\rangle }_{\mathrm{data}}-{\langle v_{i}h_{j}\rangle }_{\mathrm{model}}\right)$$

(6) $$\Delta b_{i}=\epsilon \left({\langle v_{i}\rangle }_{\mathrm{data}}-{\langle v_{i}\rangle }_{\mathrm{model}}\right)$$

(7) $$\Delta C_{i}=\epsilon \left({\langle h_{j}\rangle }_{\mathrm{data}}-{\langle h_{j}\rangle }_{\mathrm{model}}\right)$$

Here, ϵ represents the learning rate, ${\langle .\rangle }_{\mathrm{data}}$, and ${\langle .\rangle }_{\mathrm{model}}$ represent the expected value of data and model, respectively.

### 3.3. Deep Belief Networks (DBN)

**Figure 6:**
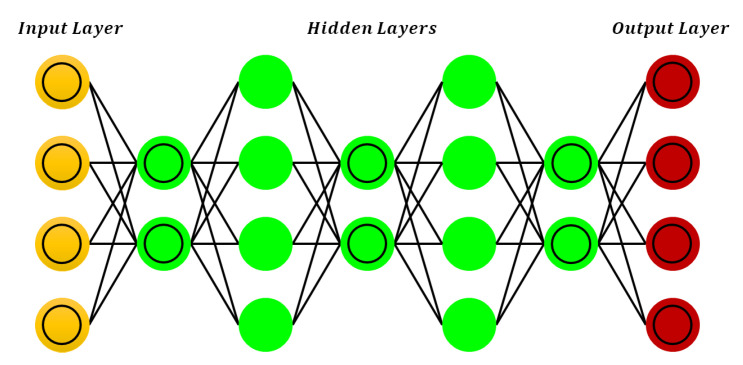
A general architecture of the Deep Belief Networks (DBN).

DBNs are made up of several layers of latent variables. These variables are typically binary and indicate the hidden characteristics of the input observations. DBN with one layer is the same as an RBM because the connection among the two top layers is undirected [31]. The remaining connections in DBN have directed graphs towards the input layer. A DBN model follows a top-down approach to generate a sample [32]. First, the samples are drawn from the RMB on the top layer using Gibbs sampling. After that, simple execution of ancestral sampling is performed by visible units in a top-down approach. A generic DBN model is presented in Figure 6.

The inference in DBN is an uncontrolled issue due to explaining away effect in the latent variable model. Hin presented an efficient and fast method to train DBN by RBM stacking: at the initial stage, the lowest level of RBM learns the distribution of input data during the training process [33]. At the next stage, RBM computes the higher-order correlation among the hidden units of the previous layer. The entire process is repeated for each hidden layer. At the next stage, RBM computes the higher-order correlation among the hidden units of the previous layer. The entire process is repeated for each hidden layer. A DBN with *Z* number of hidden layers models the joint distribution among the visible layer *v* and hidden layer $h_{z}$, here z=1,2,3,…,Z as described in the following.

(8) $$p\left(v,h_{1},\ldots ,h_{z}\right)=p\left(v|h_{1}\right)\left(\prod_{z=1}^{z-2}p\left(h_{z}|h_{z+1}\right)\right)p\left(h_{z-1},h_{z}\right)$$

Hinton’s training technique led directly to the modern era of DL, as it was the first deep architecture that had been trained efficiently. By adding layers to the NN, the logarithmic probability of the training data can be increased greatly. The DBN has been used as a classifier in several applications, such as computer recognition, phone recognition, etc. In speech recognition, DBN is used for pre-training of the DNN, deep convolutional neural network, and many others.

### 3.4. Autoencoders (AE)

**Figure 7:**
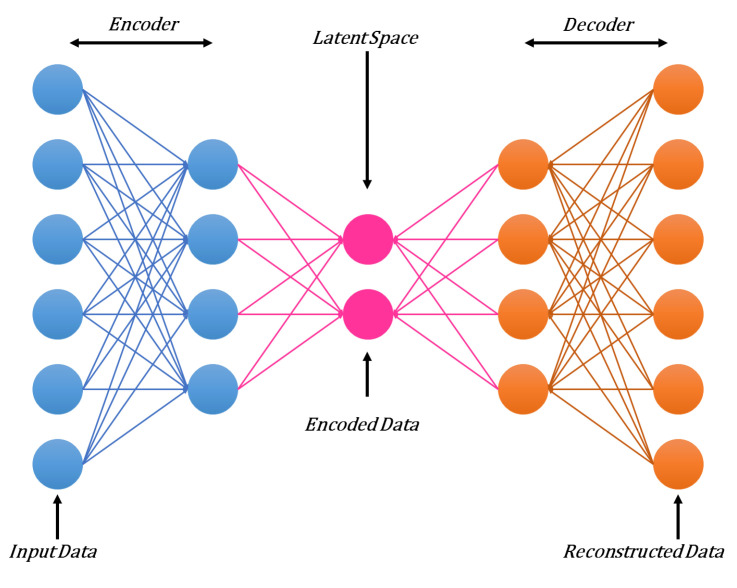
A general architecture of an Autoencoder (AE).

An autoencoder is an unsupervised learning technique for neural networks that trains the network to ignore noise signals and thus develop more efficient data representations. AE is a 3-layer neural network, as presented in Figure 7. Here the input and output layers have the same number of units, but the hidden layer usually contains a lower number of neurons. The hidden layer encodes the input data into a compact form. AE generally uses deterministic distributions instead of particular distributions, as in the case of RBM [34]. A backpropagation algorithm is usually used to train an AE. This training process contains two stages: encoding and decoding. In the first stage, the model encodes the input into hidden representations through the weight metrics. In the decoding phase, the model reconstructs the same input from an encoded representation using the weight metrics. The encoding and decoding process can be further elaborated mathematically, as presented in Equations (9) and (10).

In the encoding stage.

(9) $$y^{\prime }=f\left(wX+b\right)$$

Here, *X* represents an input vector, *f* is an activation function, *w* and *b* are the parameters that are required to be tuned and *y* is the hidden representation.

In decoding stage

(10) $$X^{\prime }=f\left(w^{\prime }y^{\prime }+c\right)$$

Here *X* represents the reconstructed input at the output layer, $w^{\prime }$ is the transpose of *w* and *c* represent bias value to the output layer.

(11) $$w_{new}=w-\eta \frac{\partial E}{\partial W}$$

(12) $$b_{new}=b-\eta \frac{\partial E}{\partial b}$$

Here, $w_{new}$ and $b_{new}$ are the updated parameters after the completion of the current iteration. *E* represents the reconstruction error at the output layer.

A deep autoencoder (DAE) is composed of multiple hidden layers of AE. Because of multiple layers, the training of AE can be a difficult task [35]. This difficulty can be overcome by training each layer of a DAE as simple AE. The DAE has been successfully applied in several applications, such as document encoding for faster subsequent retrieval, speech recognition, and image retrieval, etc. [36]. AEs gained great attention from different researchers because of their non-generative and non-probabilistic nature.

### 3.5. Convolutional Neural Network (CNN)

CNNs are a type of neural network inspired by the human visual system. CNNs were initially proposed by LeCun in 1998 [37] and gained popularity in the DL frameworks when Krizhevsky et al. [38]. won the ILSVRC-2012 competition with his proposed architecture AlexNet. This remarkable achievement started a new trend in AI, as data scientists observed the great classification capabilities of CNN and its variants. In many applications, CNN algorithms proved their superior performance in human recognition systems.

**Figure 8:**
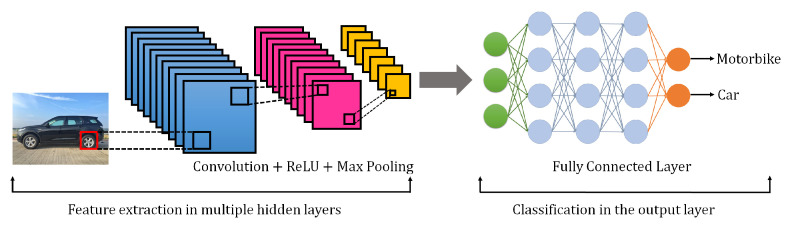
A general architecture of the Convolutional Neural Network.

The fundamental architecture of CNN is presented in Figure 8. It contains multiple convolutional and pooling layers and a fully connected layer at the end. The convolutional layer’s primary role is to extract essential features from the provided input image based on the spatial relationship between pixels. Pooling layers perform dimensionality reduction of feature maps while maintaining feature information. Finally, a fully connected layer connects the neural network with the output layer to obtain the desired output. CNNs are very useful for image descriptor extractions using latent spatial information. A common image contains several characteristics such as color, contours, edge, textures, strokes, gradient, and orientation. A CNN splits an image according to the aforementioned properties and learns them as representation in various layers. CNNs gained great popularity in computer vision applications such as image detection, image classification, image segmentation, and image super-resolution reconstruction. Multiple CNN frameworks have been proposed by considering the real-world application requirements and maintaining the high accuracy threshold. Region-based CNN (R-CNN) and You Only Look Once (YOLO) are popular examples of such modern architectures. The common naive-based CNNs are computationally very expensive because they consider a huge number region proposal to find an object within an image. However, R-CNN-based addresses this issue by the selection of a region of interest (ROI) with a selective search. Redmon et al. [39] initially proposed a YOLO technique in 2016. It has very high speed as compared to R-CNN with little compromise in performance. It understands the generalized representation of an image by looking at the object once with a CNN. However, this technique faces spatial constraints in the detection of smaller objects. Apart from these frameworks, there are several other variants of CNN have been proposed, such as AlexNet, LeNet, VGGNet, ResNet, GoogleNet, ZFNet, and many others [12]. These CNN frameworks have had a great impact on AI-enabled vision research for future applications.

### 3.6. Recurrent Neural Network (RNN)

An RNN is a class of ANN in which connections between nodes make a directed graph along a temporal sequence [40]. This property allows it to exhibit temporal dynamic behavior. In conventional neural networks (CNNs), all inputs and outputs are independent of one another. In some cases, when it is required to predict the next word or statement, then previous data are required. Therefore, there is a need to remember the previous data. RNN addresses this issue with the help of a hidden layer. The most significant feature of an RNN is the hidden state that remembers the sequence information [40]. RNN has a memory that recollects all the information about what has been calculated during the process. It uses the same parameters for all inputs and the information about what has been calculated during the process. It uses the same parameters for all inputs and performs the same tasks on all the input or hidden layers to generate a suitable output. As compared to other neural networks, this feature reduces the complexity of parameters.

An RNN is a class of ANN in which connections between nodes make a directed graph along a temporal sequence [41]. This property allows it to exhibit temporal dynamic behavior. In conventional neural networks, all the input and outputs are independent of each other. In some cases, when it is required to predict the next word or statement then previous data are required. Therefore, there is a need to remember the previous data. RNN addresses this issue with the help of a hidden layer. The most significant feature of RNN is the hidden state that remembers the sequence information [42]. RNN has a memory that remembers all the information about what has been calculated during the process. It uses the same parameters for all inputs and performs the same tasks on all the input or hidden layers to generate a suitable output. As compared to other neural networks, this feature reduces the complexity of parameters.

**Figure 9:**
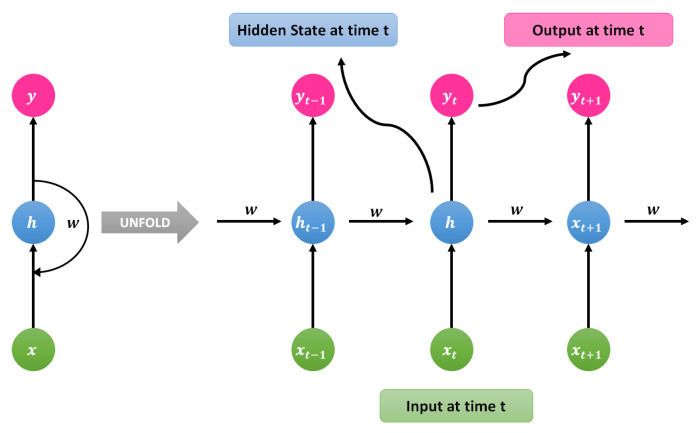
A general architecture of the Recurrent Neural Network (RNN).

The general architecture of RNN is shown in Figure 9. To understand the working mechanism of RNN, we describe a brief example here. Suppose there is a DNN that contains 1 input layer, 3 hidden layers, and 1 output layer. Each layer will have its own set of weights and biases. Let us assume for hidden layers the weights and biases for each layer is described as $w_{1},w_{2},w_{3}$ and $b_{1},b_{2},b_{3}$. Each of these layers is independent of the others as they do not memorize the previous output. RNN converts the independent activations into dependent activations by providing the same biases and weights to each layer. It memorizes each of the previous outputs as input to the next hidden layer, which results in complexity reduction. Here, these 3 layers can be converted into a single recurrent layer in such a way that the weights and biases are identical for all the hidden layers. The existing state can then be calculated using the following expression.

(13) $$h_{t}=f\left(h_{t-1},x_{t}\right)$$

Here $h_{t}$ represents the current state, $h_{t-1}$ is the previous state and $x_{t}$ is the input state of the neural network.

Now a hyperbolic tangent (tanh) activation function can be applied through the following expression.

(14) $$h_{t}=\tanh\left(w_{hh}h_{t-1}+w_{xh}x_{t}\right)$$

Here $w_{hh}$ is weight at current neuron and $w_{xh}$ is weight at input neuron.

Now the output can be calculated using the following expression.

(15) $$y_{t}=w_{hy}h_{t}$$

Here $y_{t}$ represents the output and $w_{hy}$ is the weight at the output layer.

### 3.7. Generative Adversarial Networks (GAN)

GANs are algorithmic frameworks that employ two neural networks to generate new and synthetic data instances that can pass for real data. GANs are used widely in voice, image, and video generation applications [43]. Ian Goodfellow initially introduced GAN in 2014 at the University of Montreal, Canada. Yann LeCun, the AI research director of Facebook, has called adversarial training the most emerging idea in the last 10 years of ML. GANs have great potential in multiple applications because of their ability to mimic any kind of data distribution [44]. These networks can also be trained to create worlds similar to our own choice in several domains, such as speech, music, image, video, etc. In a sense, these networks are considered robot artists that can produce impressive output. GANs can also be used to generate fake media content, a technology that has come to be known as Deepfakes.

**Figure 10:**
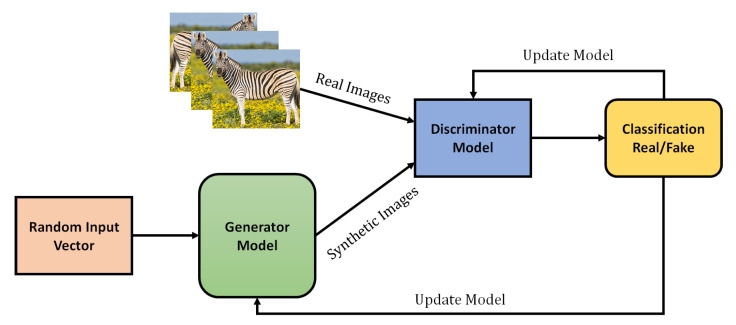
A general architecture of the Generative Adversarial Networks (GAN).

GANs are made up of two models. The first model is referred to as a generator, and its primary function is to produce new data that is comparable to the desired data. The generator might be compared to a human art forger, who makes counterfeit pieces of art. The second model is called a discriminator. This model validates the input data, deciding whether it is from the original dataset or was created by a forger. In this context, a discriminator is comparable to an art expert who attempts to determine if artworks are genuine or fraudulent. The working process of GANs are presented in Figure 10. The GAN can be easily understand using universal approximators such as ANN. A generator can be model using a neural network $G\left(n,\theta_{1}\right).$ Its main function is to map input noise variables *n* to the desired data space *x*. Conversely, the discriminator can be model using a second neural network $D\left(x,\theta_{2}\right)$. It generates the output probability of data authenticity in the range of (0,1). In both cases, $\theta_{i}$ describes the weights of each neural network.

Consequently, the discriminator trains to categorize the input data accurately, which means that the weights of neural networks are updated to optimize the probability that any real data input *x* is categorized as belongs to a real dataset. The generator is trained to generate the data as realistically as possible. This also means that the weights of the generator are tuned to increase the probability that any fake image is classified as part of the original dataset. After multiple executions of the training process, the generator and discriminator will reach the point where further improvement is not possible. At this point, the generator produces realistic data synthetic data, and the discriminator is unable to differentiate between the two types of input. During training, both the generator and discriminator will try to optimize the opposite loss function [45]. The generator tries to maximize its probability of having its output recognized as real, while the discriminator tries to minimize this value.

## 4. Deep Learning Frameworks

**Figure 11:**
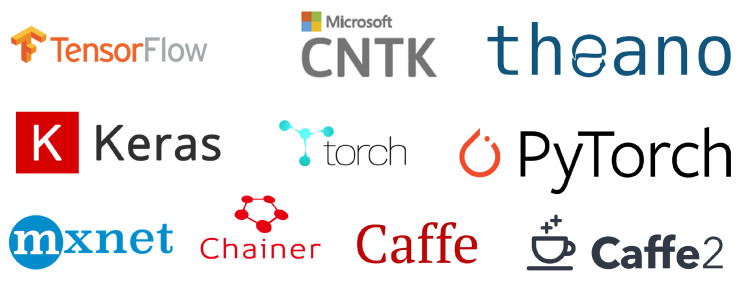
Some well-known ML/DL frameworks.

Several ML and DL frameworks are already facilitating the users to use the graphical processing unit (GPU) accelerators and thus expedite the learning process with interactive interfaces. Some of these frameworks allow for the use of optimized libraries, such as OpenCL and CUDA, to further enhance the performance. The most significant feature of multicore accelerators is their significant ability to expedite the computations of matrix-based operations [46]. The software development in ML/DL is highly dynamic and has multiple layers of abstraction. The most commonly known ML/DL frameworks are presented in Figure 11. A short description of each framework, along with its advantages and disadvantages, is described in the following.

### 4.1. TensorFlow

This is an open-source library for numerical calculations using data flow graphs [47]. This framework was created and maintained by the Google Brain team at Google’s Machine Intelligence research organization for ML and DL, and it is designed especially for large-scale distributed training and inference. The nodes in the network represent mathematical processes, while the graph edges define the multidimensional data arrays transferred between nodes. TensorFlow’s framework is made up of distributed master and slave services with kernel implementations. This framework contains around 200 operations, such as arithmetic, array manipulation, control flow, and state management. TensorFlow was designed for both research and development applications. It can easily execute on CPUs, GPUs, mobile devices, and large-scale systems with hundreds of nodes [48]. Additionally, TensorFlow Lite is a lightweight framework for embedded and mobile devices [49]. It facilitates the on-device ML/DL inference with low latency and small binary size. It supports multiple programming interfaces that include API for C++, Python, GO, Java, R, Haskell, etc. The TensorFlow framework is also supported by Amazon and Google cloud environments.

### 4.2. Microsoft CNTK

Microsoft Cognitive Toolkit is a commercial-grade distributed DL framework that includes large-scale datasets from Microsoft Research [50]. It facilitates the implementation of DNN for image, speech, text, and handwritten data. Its network is defined as a symbolic graph of vector operations using building blocks. These operations include matrix addition/multiplication or convolution. CNTK supports RNN, CNN, and FFNN models and enables the implementation of stochastic gradient (SGD) algorithms with automatic parallelization and differentiation across multiple servers [51]. CNTK is supported by both Windows and Linux operating systems using C++, C#, Python, and BrainScript API.

### 4.3. Keras

This is a Python library that enables bindings between several DL frameworks such as Theano, CNTK, TensorFlow, MXNet, and Deeplearning4j [52]. It was released under the MIT license and was developed especially for fast experimentation. Keras runs on Python 2.7 to 3.9 and can execute smoothly on both CPUs and GPUs. Francois Chollet developed and maintained this framework under four guiding principles [53], namely:

1. It follows the best practices by offering simple, consistent APIs to reduce cognitive load.
2. Neural layers, optimizers, cost functions, activation functions, initialization schemes, and regularization methods are all separate modules that can be combined to construct new models.
3. The addition of new modules is easy and existing modules provide sufficient examples that allow for the reduction of expressiveness.
4. It works with Python models that are easy to debug as well as compact and extensible.

### 4.4. Caffe

This framework was developed by Yangqing Jia and community contributors at the Berkeley Artificial Intelligence Research (BAIR) lab [54]. It provides speed, expression, and modularity. In Caffe, DNN are defined layer by layer. Here, a layer is the fundamental unit of computation essence of a complete model. Accepted data sources include Hierarchical Data Format (HDF5), efficient databases (LMDB or LevelDB), or popular image formats (e.g., JPEG, PNG, TIFF, GIF, PDF). In this framework, common layers provide normalization and vector processing operations. It provides Python support for custom layers but is not very efficient in doing so. Therefore, the new layers must be written in C++ CUDA [55].

### 4.5. Caffe2

At Facebook Yangqing Jia developed Caffe2, which is a modular, lightweight, and highly scalable DL framework [56]. Its major goal is to provide a fast and simple approach that promotes DL experimentation and exploits research contributions of new models and techniques. Its development is done in PyTorch, and it is used at the production level at Facebook. It has several improvements over Caffe, particularly in terms of new hardware support and mobile deployment. The operator is the fundamental unit of computation in this framework. Currently, Caffe2 has about 400 operators and several other operators are expected to be implemented by the research community. This framework provides Python scripts that facilitate the translation of existing Caffe models into Caffe2. However, this conversion procedure requires a manual verification process of accuracy and loss scores. Caffe also facilitates the conversion of the Torch model to Caffe2 [57].

### 4.6. MXNet

This DL framework is designed for both flexibility and efficiency. Pedro Domingos developed this framework with colleagues at the University of Washington, USA. This framework is licensed under an Apache 2.0 license. It has wide support for Python, R, Julia, and many other programming languages. In addition, several public cloud providers support this DL framework [58]. MXNet supports imperative programming, which improves productivity and efficiency. At its core, this DL framework contains a dynamic dependency scheduler that parallelized both imperative and symbolic operations automatically. A graph optimization layer expedites the symbolic execution make it memory efficient. It is a lightweight, portable framework that is highly compatible with multiple CPUs, GPUs, and other machines. It also facilitates the efficient deployment of trained DL models on resource-constrained mobile and IoT devices [59].

### 4.7. Torch

This is a scientific computing platform that supports a wide range of ML/DL algorithms based on the high-level programming language Lua. Many organizations such as Google, Facebook, Twitter, and DeepMind support this framework [60]. This framework is freely available under a BSD license. It uses object-oriented programming and C++ for implementation. Recently, its application interfaces have also been written in Lua, thus enabling optimized C/C++ and CUDA programming. Torch is built around the Tensor library, which is accessible with both CPU and GPU backends. The Tensor library is effectively implemented in C, which enables it to offer a wide range of classical operations. This DL framework supports parallelism on multicore CPUs and GPUs via OpenMP and CUDA, respectively. This framework is mostly used for large-scale learning such as image, voice, and video applications, optimization, supervised and unsupervised learning, image processing, graphical models, and reinforcement learning [61].

### 4.8. PyTorch

This is a Python library for GPU-accelerated deep learning frameworks. Facebook’s AI research department introduced this framework, which is written in Python, C, and CUDA, in 2016. It incorporates several acceleration libraries, including NVIDIA and Intel MKL. At the core level, it uses CPU and GPU Tensors and NN backend written as independent libraries [62]. Tensor computing is supported in PyTorch, with substantial GPU acceleration. It gained great popularity because of the ease with which it enables users to implement complex architectures. It also employs a reverse mode auto differentiation approach to modify network behavior with minimal effort. Industrial and scientific organizations widely use this framework. Uber created Pyro, a universal probabilistic programming language that leverages PyTorch as a backend, in a popular application. This library is supported by Twitter, Facebook, and NVIDIA and is freely available under the BSD license [63].

### 4.9. Theano

This is a pioneering DL tool that supports GPU computations and is actively maintained by the Montreal Institute for Learning Algorithms at the University of Montreal, Canada. It is also an open-source framework available under the BSD license [64]. Theano uses the NumPy and BLAS libraries to compile mathematical expressions in Python and thus transform complicated structures into simple and efficient code. These libraries enable speedy executions on CPUs and GPUs platforms. Theano has a distributed framework for training models and support extensions for multi-GPU data parallelism [65].

### 4.10. Chainer

This is an open-source, Python-based framework for DL models developed by researchers at the University of Tokyo, Japan. It offers a diverse set of DL models, including RNN, CNN, variational encoders, and reinforcement learning [66]. The main intent of Chainer is going beyond invariance. This framework enables automatic differentiation application interfaces based on the Define-by-Run technique. It creates neural networks dynamically, as compared to other frameworks. For high-performance training and inferences, this framework supports CUDA/cuDNN through CuPy. It also uses the Intel MKL library for DNN that accelerates DL schemes for Intel-based architectures. It has several libraries for industrial applications, such as ChainerRL, ChainerCV, and ChainerMN. In a study performed by Akiba in 2017, ChinerMN demonstrated its superior performance in a multi-node setting as compared to the results achieved by CNTK, MXNet, and TensorFlow [67].

## 5. Hardware Platforms for the Implementation of Deep Learning Algorithms

**Figure 12:**
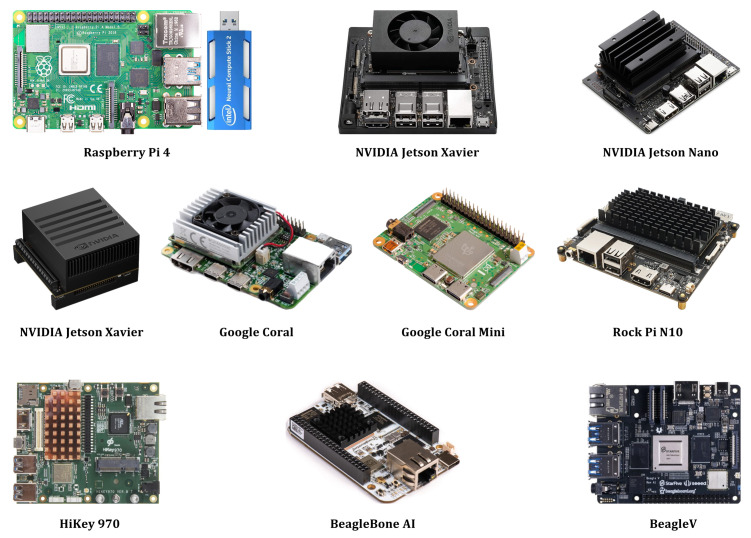
Some well-known Development Boards for DL Implementations.

In the early days of this field, special labs were established for the implementation of AI and DL techniques. These labs were equipped with high-performance computing machines that were not affordable for an individual researcher. However, with ongoing advancements in embedded electronics, single-board computers (SBC) have gained great popularity in both academia and industry for the deployment of real-time DL algorithms in industrial applications. Currently, many development boards with the capabilities of AI use are available on the market. Some well-known hardware development platforms for DL deployments are presented in Figure 12. In the following, we discuss some well-known and powerful SBCs that can or have been used for DL implementations.

### 5.1. Raspberry Pi 4

This is the latest development board of the popular Raspberry Pi family of SBC. It offers high processing speeds, large memory, efficient multimedia performance, and vast connectivity, particularly as compared to the previous generations of Raspberry Pi modules. This development board is released with a well-documented user manual that can be very helpful for beginners to expedite their learning process. The hardware specifications of Raspberry Pi 4 present the option of 2 GB, 4 GB, and 8 GB DDR4 RAMs that make this SBC an ideal choice for AI and DL-based projects [68]. Due to these characteristics, this SBC has great support from the scientific community. Raspberry Pi 4 is often used in object detection and image processing applications. Several open-source ML/DL libraries provide great support for the speedy implementation of real-world applications. This development board is also complemented by AI-supported third-party accessories. Raspberry Pi used along with Intel Neural Computing Stick 2 makes an ideal combination for the development of AI frameworks. Another alternate third-party accessory is the Coral Edge TPU USB accelerator that enables this SBC for AI-enabled applications [69]. Additional platforms, such as Google’s AIY Vision and Voice kits, can also be paired with Raspberry Pi 4 to make significant contributions in AI and DL projects.

### 5.2. NVIDIA Jetson Xavier^TM^

This platform provides supercomputer performance at edge in a compact system-on-module. It also has new cloud-native support that accelerates the NVIDIA software stack. The power and efficiency of this module enable multi-modal and accurate AI inference in a small form factor. The development of this SBC opened new doors for innovative edge devices in logistics, manufacturing, retail, agriculture, service, healthcare, smart cities, and many other applications [70]. The Jetson Xavier NX module with cloud-native support enables the design, deployment, and management of AI edge. The NVIDIA Transfer Learning Toolkit with pre-trained AI models from NVIDIA NGC provides a faster interface with optimized AI networks. To improve the performance of existing deployments, this module supports flexible and seamless updates. NVIDIA JetPack™ SDK facilitates the development of applications for Jetson Xavier NX with supporting libraries. It supports several AI frameworks such as graphics, multimedia computer vision, and many others. JetPack ensures reductions in development costs with the integration of the latest NVIDIA tools.

### 5.3. NVIDIA Jetson Nano^TM^

This module is an SoM of Jetson Nano Development Kit, and it tends to be used mostly to assemble a development board to deploy in AI-based graphic applications. This powerful AI-enabled SBC enables developers, students, and hobbyists to implement DL algorithms and models for several applications such as object detection, image classification, speech processing, image segmentation, and more [71]. This module is available with the multiple features of Jetson Nano Development Kit, which includes the main CPU, GPU, and several user interfaces such as USB, GPIO, and CSI. Instead of using a MicroSD card, the Jetson Nano module has 16 GB eMMC storage. All functionalities of this SBC can be accessed via an M.2 E connector.

### 5.4. NVIDIA Jetson AGX Xavier^TM^

Jetson AGX Xavier development board provides great support for the development of end-to-end AI-enabled robotics applications for delivery, agriculture, retail, manufacturing, and many other fields. This development board facilitates the industrial or business circumstances related to automobile upgrades, horticulture, production, manufacturing, and retailing, among others. NVIDIA designed this SBC especially for advanced- level developers and engineers to explore the great potential of AI deployments in industrial, scientific, and business applications. The advanced capabilities of NVIDIA Jetson AGX Xavier make it an ideal choice for companies, businesses, and industries seeking to create versatile applications [72].

### 5.5. Google Coral

When a deployment of rapid ML/DL inferences in a compact form factor is required, this development board is an appropriate choice. This SBC can be used to prototype the embedded system and subsequently scale to production using the onboard Coral SoM combined with custom hardware. Google Coral provides a fully integrated system that includes NXP’s iMX 8M, LPDDR4 RAM, eMMC memory, Bluetooth, and WiFi. Its distinct processing capability is provided by Google’s Edge TPU co-processor. Google created the Edge TPU, a tiny ASIC architecture that offers high-performance ML/DL inferences while consuming relatively little power. It can execute advanced mobile vision models such as MobileNet v2 at almost 400 FPS, all in an energy-efficient manner [73].

### 5.6. Google Coral Dev Board Mini

This is a low-cost SBC with a built-in Edge TPU and real-time inference module for powerful ML/DL algorithms. This development board integrates MediaTek 8167 SoC with the Edge TPU, making it a standalone hardware platform capable of executing AI applications smoothly. TensorFlow Lite can be used with Edge TPU for quick and energy-efficient inferences. This integration provides adequate data protection according to the General Data Protection Regulation (GDPR) [74]. Google uses ML/DL-based solutions in its services at a large scale. Google introduced specialized processors TPU that can execute ML/DL algorithms in a faster and energy-efficient manner using TensorFlow. The best example is Google Maps Street view, which is analyzed by a TensorFlow-based neural network. Edge TPU supports TensorFlow Lite, which is highly compatible with embedded and mobile devices for efficient ML/DL implementations [74].

### 5.7. Rock Pi N10

This development board is a new member of the Rock Pi family. It contains a powerful SoC RK3399Pro that is integrated into GPU, CPU, and NPU. RK3399Pro’s CPU is a six-core that includes Dual Cortex-A72 and quad Cortex-A53. Its GPU supports Open CL 1.1 1.2, Vulkan, OpenGL ES 1.1/2.0/3.0/3.1/3.2, and DXI. Its NPU supports 8/16bit computing. The NPU has very good performance for complex calculations, which can be very helpful in DL applications [75]. This development board has multiple resources for storage. A 64 GB eMMC 5.1 and 64 bits dual-channel 8 GB LPDDR are embedded on the mainboard. Rock Pi N10 also supports multiple interfaces for camera, audio, USB, Ethernet, display, and I/O pins. This development board uses Android 8.1 and Debian as its firmware [75].

### 5.8. HiKey 970

This is a Super-Edge AI computing platform that is supported by Kirin 970 SoC with 4 × Cortex A53 and 4 × A73 processors. It has 6 GB UFS storage, 6 GB LPDDR4 RAM, and Gigabit Ethernet onboard. It is considered the world’s first dedicated NPU AI platform, which integrates popular neural network frameworks and Huawei HiAI computing architecture [76]. It supports CPU, GPU, and NPU all dedicated to AI acceleration. This SBC is mostly used to implement DL algorithms in automobiles, robots, and smart city applications [76].

### 5.9. BeagleBone AI

This development board provides great support for the use of AI in everyday applications. It has embedded vision engine (EVE) cores and the T1 C66x digital signal processor cores supported by OpenCL API with pre-installed tools. It is widely used in home automation as well as commercial and industrial applications [77]. This SBC is considered an ideal candidate for AI and DL implementations. It contains a rich collection of features and mimics the Linux approach, which makes it as an open-source platform. Some powerful specifications of this SBC make it more powerful than other development boards, such as its a Dual ARM Cortex A-15 processor, 2 C66x floating-point VLIW DSPs, dual-core PowerVR SGX544 3D GPU, and the dual-core programmable real-time unit, which consists of four embedded vision engines [77].

### 5.10. BeagleV

This development board offers open-source RISC-V computing on Linux distributions such as Fedora and Debian. This is a powerful SBC that contains a SiFive U74 processor, 8 GB RAM, and multiple I/O ports. BeagleV is comparatively affordable, as contrasted with other SBCs. The SiFive U74 processor contains two cores and an L2 cache that can all compete with the performance of other processors, such as ARM Cortex-A55 [78]. The SiFive U74 also includes a Vision DSP Tensilica-VP6, an NVDLA Engine, a Neural Network Engine, and an audio processing DSP. Currently, this development board is only available without GPU, but according to CNX software, it will also be available with GPU from September 2021. This SBC facilitates developers’ ability to extend their projects more strongly and flexibly.

## 6. Applications of Deep Learning in the IIoT

DL techniques have shown promising results in several applications. This section discusses some potential use cases of DL in IIoT applications.

### 6.1. Agriculture

**Figure 13:**
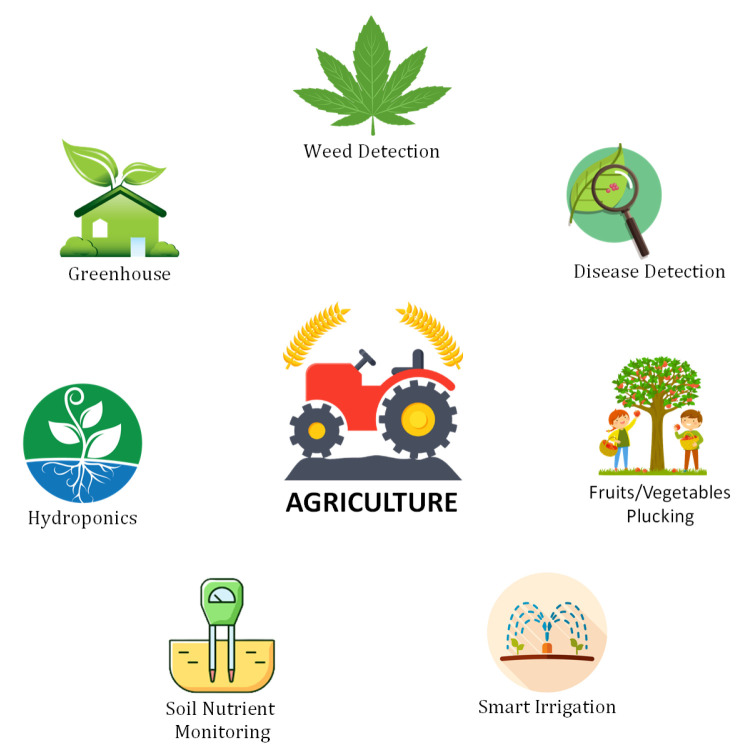
Applications of DL in agriculture.

Agriculture is a major industry and foundation of the economy in several countries. However, multiple factors such as population growth, climate change, and food safety concerns have propelled researchers to seek more innovative techniques for the protection and improvement of crops. In developed economies, AI and DL techniques are frequently used for improving the productivity and quality of agricultural products. Some popular research contributions in the context of DL techniques for the agricultural industry can be found here [79,80,81,82,83,84,85,86,87,88]. A comparison of some prominent studies is presented in Table 2. In the following, we discuss several prominent applications of DL in the agricultural sector as shown in Figure 13.

#### 6.1.1. Weed Detection

When a vast number of data are produced by certain applications, then DL techniques are considered to be an ideal choice of solution. In weed detection problems, DL models can be trained using thousands of images of weeds. After optimized training, the DL model can successfully distinguish weeds from crops.

#### 6.1.2. Smart Greenhouse

Greenhouses enable independent climate inside a specific area. It is quite complex to manage the micro-climate for a greenhouse. In this context, several environmental factors must be managed, such as temperature, humidity, wind direction, the intensity of light, heating, ventilation, etc. The use of DL methods in the greenhouse can be worthwhile to predict these outcomes and control these variables. DL can efficiently manage the inside climate by considering all the internal and external environmental factors.

#### 6.1.3. Hydroponics

This system enables the growth of plants in a nutrient-rich environment instead of soil. Several factors affect the performance of the hydroponic system, such as temperature, sunlight, pH balance, and nutrition density. DL models can be trained according to these environmental parameters to launch an appropriate control action.

#### 6.1.4. Soil Nutrient Monitoring

Soil fertility or nutrient level is determined by the organic matter in the soil, and it determines the yield and quality of an agricultural product. DL frameworks can be used to predict the presence of organic matter for the calculation of soil fertility. As a result, farmers can more easily predict the productivity and health of the soil on their farms.

#### 6.1.5. Smart Irrigation

A DL-based framework for a plant recognition system can easily determine the water requirement for a particular plant type. It can also control the amount of water flow according to the plant’s requirements. In this particular application, the convolutional neural network (CNN) can be an ideal choice, since it can recognize the plant type after training by on datasets of plant images.

#### 6.1.6. Fruit and Vegetable Plucking

Picking ripe crops requires labor and the ability to distinguish targets accurately. A DL model can be trained using image datasets of fruit and vegetables and then deployed in fruit and vegetable-picking tools such as robots. This robot can easily distinguish the specific fruit or vegetable from other objects and then pluck it. For this particular application, CNN can be an ideal choice.

#### 6.1.7. Early Detection of Plant Diseases

DL frameworks can also be used for the early-stage detection of plant diseases. In this particular application, an image dataset will be required that contains the images of plant leaves with a particular disease. A deep convolutional neural network can then be trained to identify this disease and perform this task.

### 6.2. Education

**Figure 14:**
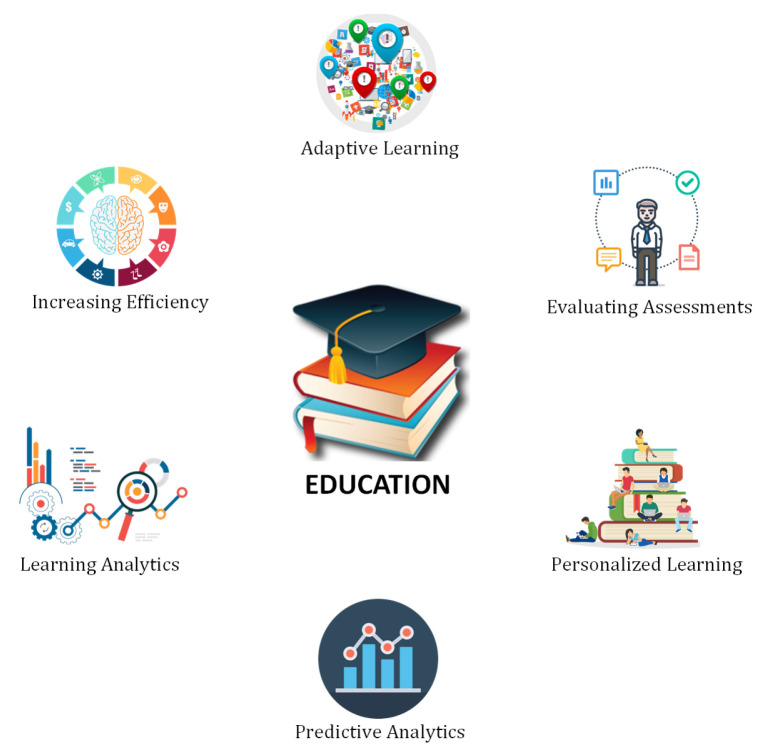
Applications of DL in education.

DL is a type of individualized learning in the education industry. It can be used to provide an individualized educational experience to each student. Here, the students are guided for their own learning, can follow the pace they want, and make their own decisions about what to learn. Some state-of-the-art research contributions in the context of DL techniques for the education industry can be found here [89,90,91,92,93,94]. A comparison of some prominent studies is presented in Table 3. In the following, we discuss some top applications of DL in the education sector as shown in Figure 14.

#### 6.2.1. Adaptive Learning

It helps the students to analyze their real-time performance and modifies teaching methodologies and curriculum according to the collected information. DL models can assist to have a customized engagement and tries to adapt to the individual for a better education. DL frameworks can suggest better learning paths to students using different materials and methodologies.

#### 6.2.2. Increasing Efficiency

DL has a great capability to organize and manage better content and curriculum. It helps to understand the potential of everyone and bifurcate the work accordingly. It can efficiently analyze that which work is best suitable for teacher and student. DL can make instructors’ and students’ jobs simpler, making them more comfortable with their educational commitments. These modern techniques can motivate the students to increase their involvement and towards participation and learning. The DL frameworks also have the great potential to make education more efficient by classroom management and scheduling etc. in summary the DL methods can significantly increase the efficiency of the education sector.

#### 6.2.3. Learning Analytics

Several times teachers can also become stuck while teaching. Because of this, the insights and gist are not properly understood by the students. Learning analytics can help the teacher to perform deep dives into the data. The instructor can go through hundreds of pieces of material, interpret them, and then draw appropriate connections and conclusions. It can make a very positive impact on the learning and teaching process. DL methods can help the students to gain benefits by learning advanced methodologies through learning analytics.

#### 6.2.4. Predictive Analytics

Predictive analytics is helpful to understand the student’s needs and their behaviors in the education sector. It helps in drawing inferences about what could happen in the future. With the use of class assessments and half-year results, it is possible to predict which students will show the best performance in the exams and which students will face difficulties. It also assists teachers and parents in being aware and taking appropriate measures. In short, predictive analysis can help the students in a better way and can also work on their weak points in education.

#### 6.2.5. Personalized Learning

This is one of the best applications that deep learning provides for education. Students can define their own learning ways through this educational model. They can study at their own pace and choose what and how they want to learn. They can select the subjects they want to study, the teacher, standards, curriculum, and pattern they want to follow.

#### 6.2.6. Evaluating Assessments

DL frameworks can be used to grade student’s assignments and examinations more accurately as compared to the human. Although some inputs are necessarily required from humans. However, the best results will have more reliability and validity when a machine performs the task with more efficiency, higher accuracy, and low chances of error.

### 6.3. Healthcare

**Figure 15:**
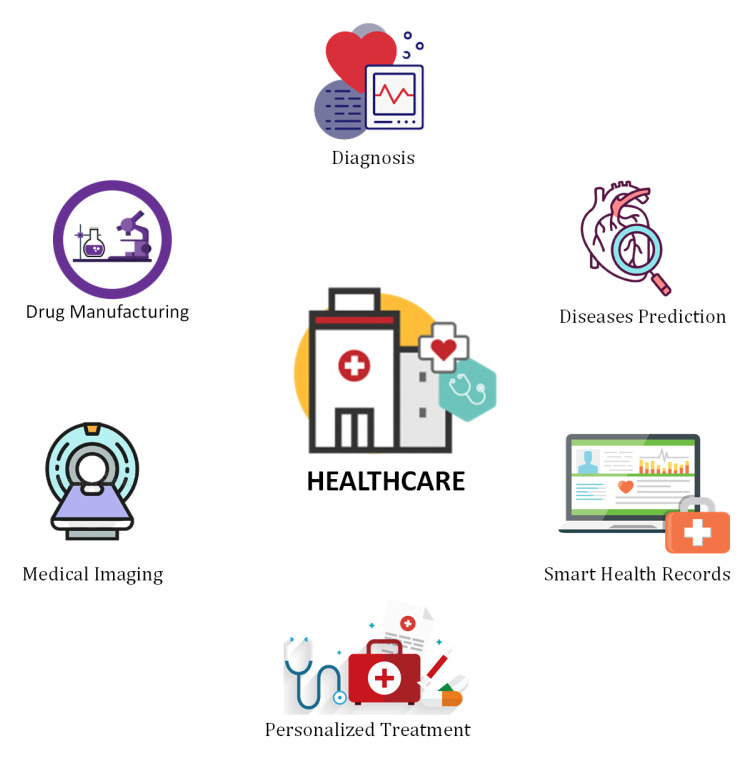
Applications of DL in healthcare.

Healthcare is an important sector that provides value treatment to millions of people while also being a top income producer for many countries. Technology is helping healthcare experts to establish alternate staffing models, IP capitalization, deliver smart healthcare, and reduce administrative and supply expenses, in addition to playing a vital role in patient care, billing, and medical records. Deep learning is gaining great attention from healthcare industries. DL can help to analyze millions of data points, precise resource allocation, provide timely risk sources, and many other applications. Some latest research contributions in the context of DL techniques for the healthcare industry can be found here [95,96,97,98,99,100,101,102,103,104,105,106,107]. A comparison of some prominent studies is presented in Table 4. In the following, we discuss some top applications of DL in the healthcare industry as shown in Figure 15.

#### 6.3.1. Diseases Identification and Diagnosis

DL can play a significant role in a patient’s disease identification, monitoring his real-time health condition, and suggest necessary measures to prevent it. It can efficiently identify the minor diseases as well as the major ones such as cancer detection which is difficult to identify at early stages. It also provides suitable information that enables more accurate diagnosis, improved outcomes, and individualized treatments.

#### 6.3.2. Drug Discovery and Manufacturing

Next-generation sequencing and precision medicine are two research and development technologies that can help in the treatment of a wide range of health problems. DL algorithms can identify the patterns in medical data without providing any predictions. Discovering or producing a novel medication may be a costly and time-consuming procedure because many chemicals are tested and only one outcome might be beneficial. In this context, DL techniques can expedite this process.

#### 6.3.3. Medical Imaging

DL algorithms have made it feasible to detect microscopic abnormalities in scanned images of patients, allowing physicians to make an accurate diagnosis. Traditional procedures such as X-ray and CT scans were sufficient for inspecting small abnormalities, but with the increasing diseases, there is a need to check them thoroughly. DL made advanced contributions in the field of computer vision. This technique was deployed in Microsoft’s Inner-Eye project, which develops image analysis technologies. This research project uses DL algorithms to provide novel tools for the automated, quantitative interpretation of 3D radiological images. This project also helps the experts in the field of surgical planning and radiotherapy.

#### 6.3.4. Personalized Medicine/Treatment

Doctors can now provide personalized treatment to individual patients based on their specific needs according to the explosion of patient data in the form of electronic health records. They aim to extract insights from enormous volumes of datasets and use them to make patients healthier at individual levels. DL technologies can help to suggest personalized combinations and predict the risks of disease. Watson healthcare creates powerful resources using ML and DL for the patient’s health improvement. This platform decreased the doctor’s time that was spent on making treatment decisions based on research analysis, clinical practices, and trials. This DL-based platform now offers blood cancer treatment and many other diseases.

#### 6.3.5. Smart Health Records

There are still some processes exist that take a lot of time for data entry. Maintaining every day’s health records is a time-consuming activity. In this context, DL can save time, effort, and money to maintain health records. Google’s Cloud Vision API and MATLAB’s DL-based handwriting recognition technology are frequently used for document classification. It facilitates the recording of clinical data, modernizes the workflow, and improvement of health information accuracy.

#### 6.3.6. Diseases Prediction

Multiple DL technologies are being used to monitor and predict epidemics all around the world. Scientists have access to vast amounts of data collected from satellites, websites, and social media platforms. DL assists in the collaboration of this information and the prediction of everything from small diseases to serious chronic infectious diseases.

### 6.4. Intelligent Transportation Systems

**Figure 16:**
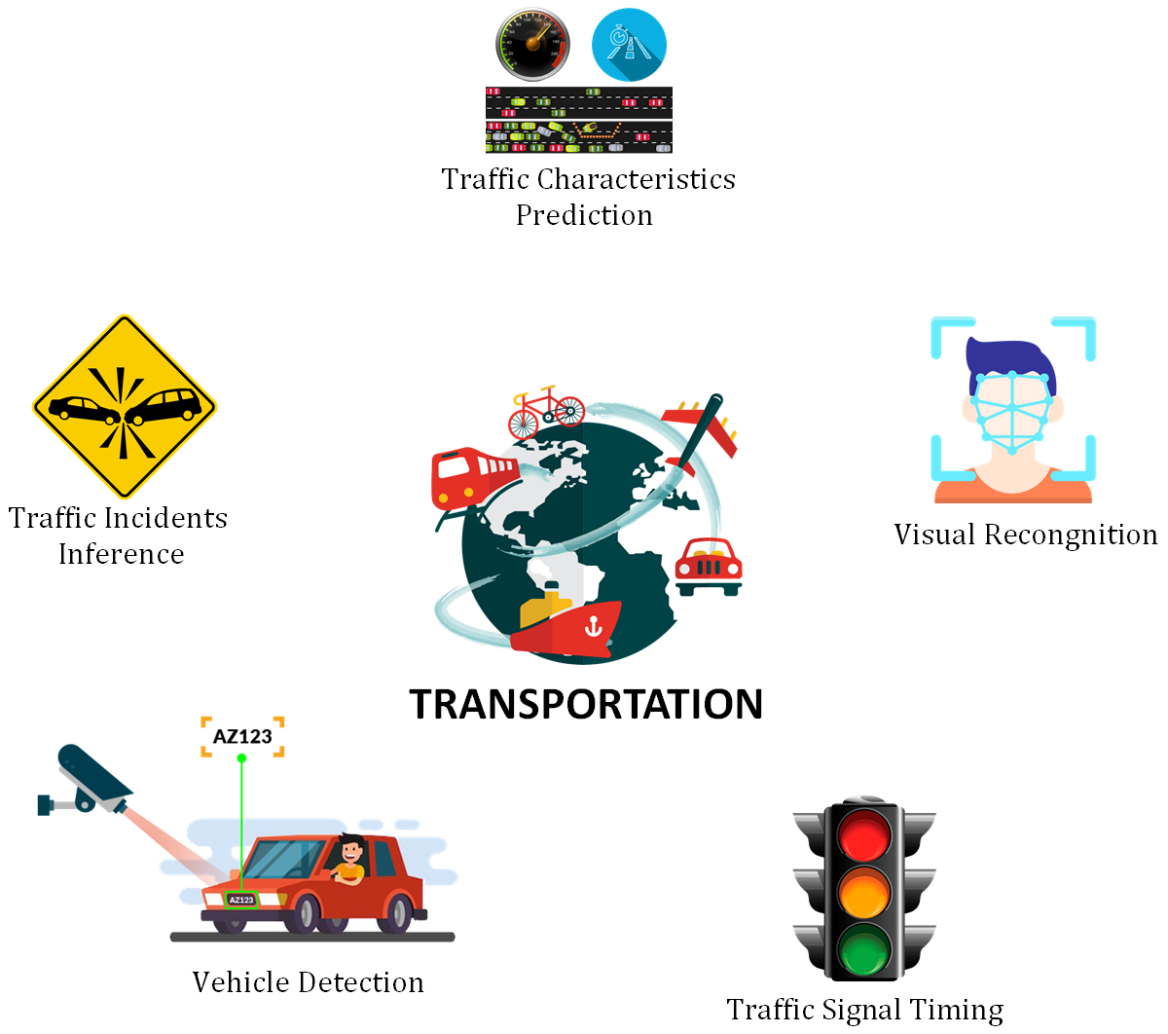
Applications of DL in intelligent transport system.

Intelligent transportation systems (ITS) have been influenced by the rapid growth of deep learning. DL gained great popularity in ITS with the proliferation of data and advancements in computational technologies. In ITS, the DL models are being used to provides powerful and viable solutions to address the multiple challenges in the traditional transport system. In the context of DL approaches, some latest research contributions related to the ITS can be found in [108,109,110,111,112,113,114,115,116,117]. A comparison of prominent studies is presented in Table 5. In the following, we discuss potential applications of DL in the ITS as shown in Figure 16.

#### 6.4.1. Traffic Characteristics Prediction

This is one of the most important applications of DL in intelligent transportation. It can help the drivers to select the most feasible routes and enable the traffic control agencies for the efficient management of transport. The main characteristics are traveling time, traffic speed, and traffic flow. As these characteristics are not mutually exclusive, however, the techniques used to predict one of them can also be used to predict the other features

#### 6.4.2. Traffic Incidents Inference

The main goal of traffic incident risk prediction for a specific location helps the traffic management authorities in reducing incident risks in a hazardous region and traffic congestion in incident locations. Several key factors can be helpful for traffic incidents prediction. These factors include traffic flow, human mobility, weather, geographical position, period, and day of the week. All these features can be evaluated as indicators of a traffic incident. DL models can be effectively used to predict and detect traffic incidents.

#### 6.4.3. Vehicle Detection

Vehicle detection in highway monitoring videos is a challenging task and very important in terms of intelligent traffic management and control. Traffic surveillance cameras are installed on highways that generate a vast amount of data in the form of traffic videos. These data can be further analyzed to identify the vehicle identification. DL algorithms provide fast and robust solutions for vehicle detection that can be helpful for traffic management authorities to ensure traffic safety on highways.

#### 6.4.4. Traffic Signal Timing

One of the major responsibilities of ITS management is to control the traffic flow using traffic signals. The optimum selection of signal timing plays a significant role in traffic flow management. This is one of the biggest challenges in the field of modern transportation. DL studies providing new paths by modeling the dynamics of traffic to obtain the best performance on the roads. In several studies, the DL algorithms proved their superior performance for the optimum selection of traffic signal timing.

#### 6.4.5. Visual Recognition Tasks

The use of non-intrusive recognition and detection systems, such as camera-image-based systems, is one of the most significant uses of DL. These applications might range from providing appropriate roadway infrastructure for driving cars to provide autonomous vehicles with a safe and dependable driving strategy.

### 6.5. Manufacturing Industry

**Figure 17:**
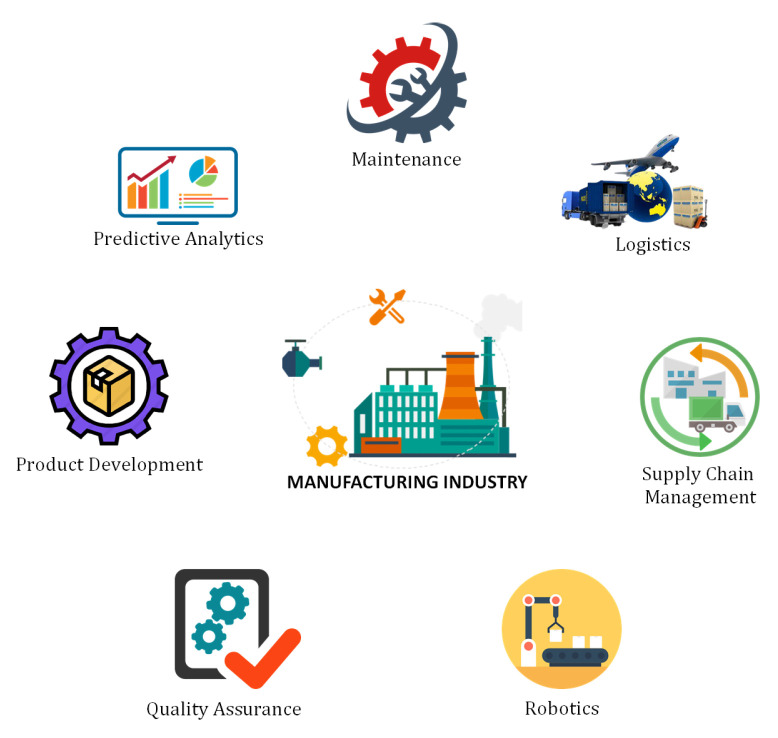
Applications of DL in manufacturing industry.

DL-based solutions are transforming manufacturing industries into highly efficient organizations by improving productivity, increasing capacity use, decreasing production defects, and reducing maintenance costs. In the context of DL approaches some latest research contributions for the manufacturing industry can be found here [118,119,120,121,122,123,124,125,126]. A comparison of some prominent studies is presented in Table 6. In the following, we discuss some potential applications of DL in the manufacturing industry as shown in Figure 17.

#### 6.5.1. Maintenance

DL algorithms are widely used for predictive maintenance in an industry to prevent failures of the machine by identifying the upcoming potential challenges more accurately. DL frameworks analyze the real-time images, sounds, and other information collected by industrial sensors to reduce the downtime of systems.

#### 6.5.2. Predictive Analytics

DL models can accurately predict operational outcomes. It enables the companies to optimize their manufacturing processes. DL algorithms use real-time sensor data monitoring production lines, inventory, waiting for the time of machines, technical and physical conditions of machines, and behavior of workers. Manufacturing companies can also examine the effectiveness of their processes by analyzing the information about quality issues, raw materials, maintenance procedures, and environmental factors such as temperature and humidity. The predictive analysis can be very helpful to identify, unprofitable lines, non-value-added activities, and bottlenecks in industrial operations.

#### 6.5.3. Product Development

DL is increasingly used in product designing. DL-based software enables the manufactures to input the important aspects of product design such as material, cost, and durability, etc. Based on the user input, this software proposes a highly suitable product design and facilitates the user for more realistic testing without having to build extremely expensive prototypes. Engineers of the automotive industry believe that the DL will be the most promising approach to design high-speed racing cars and can also be helpful to create more realistic platforms to achieve optimal performance.

#### 6.5.4. Quality Assurance

DL-enabled computer vision techniques are used for the automatic detection of defective products. Different manufacturers claim that such quality testing solutions can achieve up to 90% accuracy in defect identification for specific applications. It also enables the production teams to address the quality problems at an earlier stage. As an example, Audi uses DL-based image recognition system for the successful identification of fine cracks on metal sheets.

#### 6.5.5. Robotics

Several manufacturers use industrial robots to handle dangerous and complicated processes. Now DL frameworks enable the robots to learn on their own. A DL-enabled robot can train itself for performing new tasks using object and pattern recognition capabilities.

#### 6.5.6. Supply Chain Management

DL approaches are considered highly accurate analytical techniques that can add value in complex supply chain management operations. Using DL models companies can predict real-time demands, optimize their production schedules and supply chain operations, and can efficiently manage the inventory to reduce purchasing costs of raw materials. These capabilities of DL models enable the companies to quickly respond to the change in market demand.

#### 6.5.7. Logistics

DL models can significantly improve fuel efficiency and delivery time by analyzing the real-time information about drivers and vehicles in logistic operations.

### 6.6. Aviation Industry

**Figure 18:**
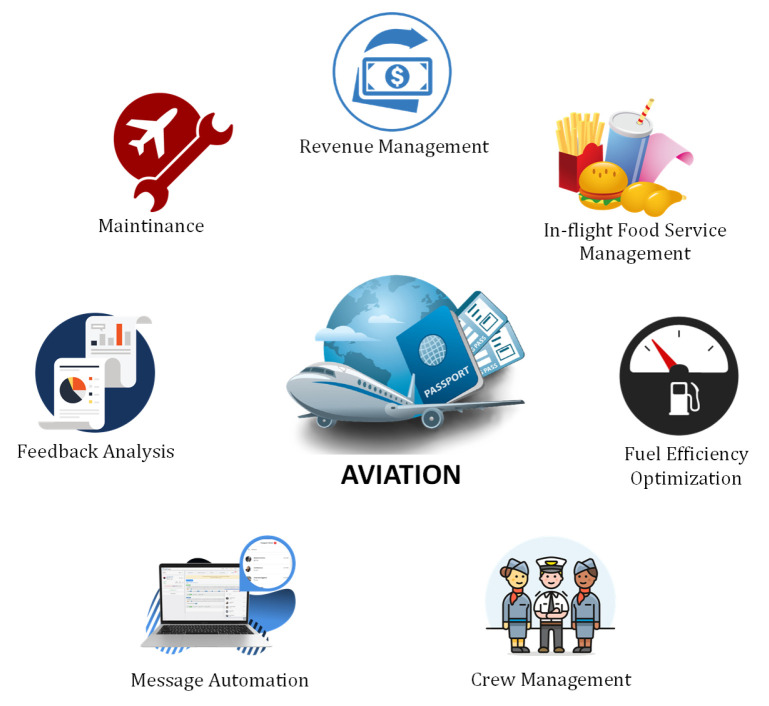
Applications of DL in aviation industry.

AI and DL can streamline and automate customer services, analytics, machinery maintenance, and many other internal procedures and operations in the aviation industry. The most recent and relevant research contributions related to the use of DL techniques in the aviation industry field can be found in [127,128,129,130,131,132,133,134,135]. A comparison of prominent studies is presented in Table 7. In the following, we discuss the most important applications of DL in the aviation industry as shown in Figure 18.

#### 6.6.1. Revenue Management

It is a popular application of data and analytics that determines the sale of an appropriate product to relevant customers at a reasonable price using the right channel. Revenue management specialists in the aviation industry use DL techniques to define attractive destinations and adjust prices, find efficient and convenient distribution channels, and seat management to keep the airline competitive and customer-friendly.

#### 6.6.2. Air Safety and Airplane Maintenance

Airlines usually bear the huge cost because of flights delays and cancellations that include expenditures on maintenance and compensations to passengers stuck in airports. According to a study, about 30% of total delay time is caused by unplanned maintenance. To address this challenge, a DL-based predictive analysis can be applied to fleet technical support. Airline carriers deploy intelligent predictive maintenance frameworks for better data management from aircraft health monitoring sensors. These systems enable the technician’s access to real-time and historical information from any location. The information about the current technical condition of an aircraft through notifications, alerts, and reports can be helpful for employees to identify the possible malfunction and parts replacement proactively.

#### 6.6.3. Feedback Analysis

AI and DL-enabled systems can swiftly assess if there is a chance to favorably intervene in the customer journey and transform a bad experience into a pleasurable one. It also enables the companies to respond rapidly in an aligned and synchronized way that is on board with the business’s values. Using DL for market research and feedback analysis enables airlines to make intelligent decisions and meet customer’s expectations.

#### 6.6.4. Messaging Automation

Travelers usually become nervous when a flight delay or baggage loss occurs. If passengers do not receive a timely response from an airline representative regarding their problem, then they will not choose this airline for their upcoming trips. The speed of response to customers matters a lot. DL-based solutions simplify and expedite the customer service processes using advanced algorithms of natural language processing. These solutions can automate several routine processes and can create ease for passengers.

#### 6.6.5. Crew Management

One of the major responsibilities of the scheduling department is to assign crews to each of thousands of flights every day. Crew management is a complex task that includes several factors such as flight route, aircraft type, crew member license, work regulations, vaccination of staff, and days off to approve conflict-free schedules for pilots and flight attendants. Several aviation companies are using AI and DL-based software to make optimal scheduling in terms of crew qualification, working hours, aircraft expenses, and use. Such software integrates predictive models with an airline operation management system.

#### 6.6.6. Fuel Efficiency Optimization

Airlines use AI frameworks with built-in DL algorithms to gather and analyze flight data regarding aircraft type, weather, weight, route distance, and altitudes, etc. DL models can efficiently estimate the optimal amount of fuel required for a specific flight.

#### 6.6.7. In-Flight Food Service Management

DL techniques can be helpful for supply management specialists to determine the number of snacks and drinks on board without being wasteful. The effective use of DL methods can predict the amount of food required for any specific flight. The optimization in foodservice management can reduce the cost and improve the service quality.

### 6.7. Defense

**Figure 19:**
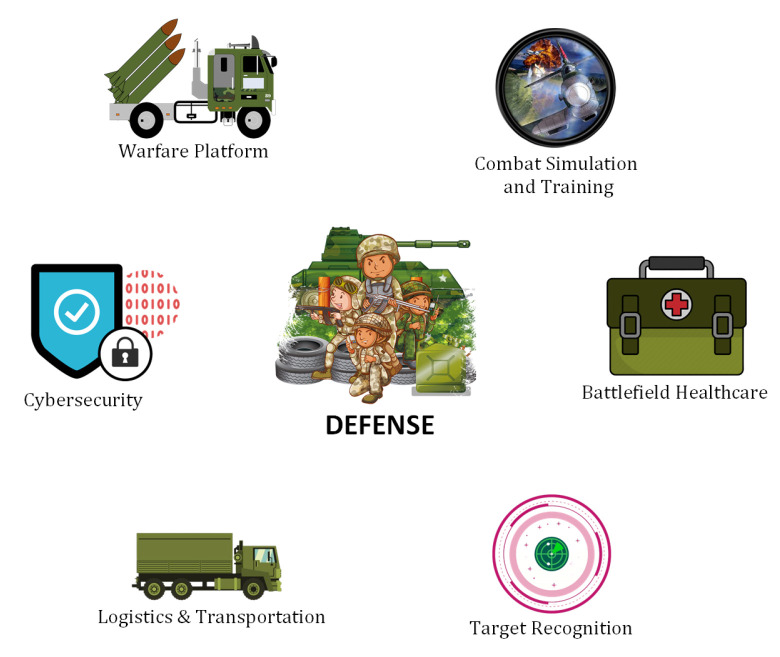
Applications of DL in defense.

ML and DL have become an essential part of modern warfare. Modern military systems integrated with ML/DL can process enormous amounts of data more effectively compared to traditional systems. AI techniques improve self-control, self-regulation, and self-actuation of combat systems because of their inherent decision-making capabilities. AI and DL are being deployed in almost every military application. Military research organizations have increased the research funding to drive the adoption of ML/DL-driven applications for military applications. Recent research contributions related to the use of DL techniques in defense and military applications can be found in [136,137,138,139,140,141]. A comparison of prominent studies is presented in Table 8. In the following, we discuss the most relevant potential applications of DL in the military domain as shown in Figure 19.

#### 6.7.1. Warfare Platforms

Armed forces from several countries across the globe are incorporating AI techniques into weapons and other military equipment used on ground, airborne, naval, and space platforms. The deployment of DL methods on these platforms has enabled the development of highly efficient autonomous warfare systems. DL enhanced the performance of warfare platforms with less maintenance.

#### 6.7.2. Cybersecurity

Military platforms are frequently vulnerable to cyber-attacks, which can result in massive data loss and damage to defense systems. However, the deployment of ML/DL techniques can autonomously protect computers, networks, data, and programs from any intrusion. Additionally, DL-enabled cybersecurity systems can record the pattern of new attacks and develop firewalls to tackle them.

#### 6.7.3. Logistics and Transportation

The efficient transportation of ammunition, goods, troops and armaments is an integral component of successful military operations. The integration of DL techniques with military transportation can reduce human operational efforts and lower transportation costs. It also enables the military fleets to quickly predict the component failures and easily detect anomalies.

#### 6.7.4. Target Recognition

ML/DL frameworks are being developed to improve the accuracy of target identification in complex combat environments. These techniques allow the armed forced to gain an in-depth understanding of critical operation areas by analyzing documents, reports, and news feeds, etc. The capabilities of DL-based target identification systems include aggregation of weather conditions, probability-based forecasts of enemy behavior, assessments of mission approaches, and suggested mitigation strategies. In addition, the integration of DL in target recognition systems improves the ability of these systems to identify the position of targets.

#### 6.7.5. Battlefield Healthcare

ML/DL can be integrated with Robotic Ground Platforms (RGPs) and Robotic Surgical Systems (RSS) to provide evacuation activities and surgical support in war zones. Under critical conditions, the health care systems equipped with ML/DL can search the soldier’s medical records and efficiently assist in complex diagnoses.

#### 6.7.6. Combat Simulation and Training

Simulation and Training is a broad field that combines software engineering, system engineering, and computer science to develop computerized models that acquaint soldiers with the various combat systems during military operations.

### 6.8. Sports Industry

**Figure 20:**
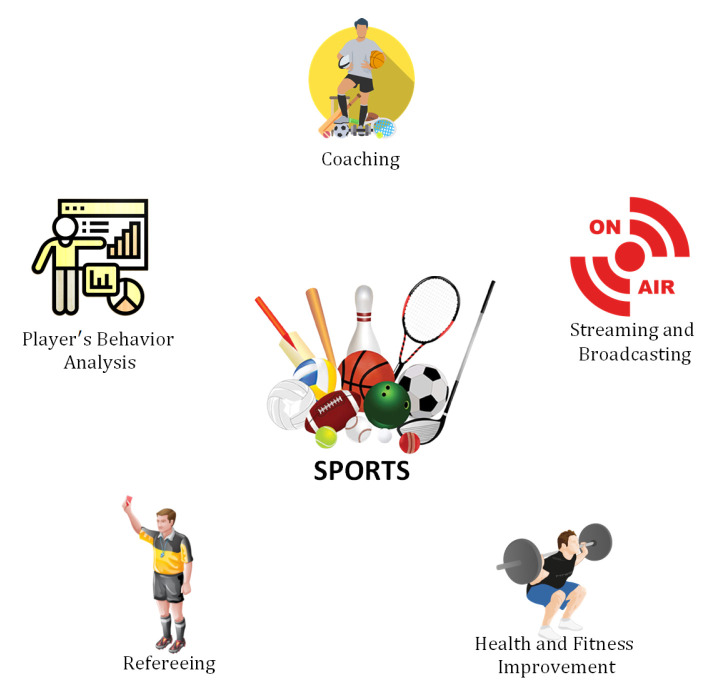
Applications of DL in sports industry.

ML and DL techniques are being adopted to better analyze, organize, and optimize every aspect of the sports industry. Management and athletes are required to gather every information about the individual and team performance to gain an edge over their opponent. AI and virtual reality techniques are increasingly used to provide real-time analytics in sports. The availability of large data in sports can be very helpful to develop predictive DL models to make better management decisions. In the context of DL approaches, the latest and relevant research contributions related to the sports industry field can be found in [142,143,144,145,146,147,148,149]. A comparison of prominent studies is presented in Table 9. In the following, we discuss relevant applications of DL in the sports industry as shown in Figure 20.

#### 6.8.1. Sports Coaching

The presence of experienced coaches is an essential factor behind every winning team. Presently, augmented AI with wearable sensors and high-speed cameras are helping a lot to improve training. The weakest point of conventional coaching is that it takes years to hone the skills. Now AI-enabled assistants are helping the coaches to improve the game strategy and optimize the team’s lineup according to modern game requirements.

#### 6.8.2. Analyzing Player Behavior

DL evaluates the multiple parameters of sports side by side. The individual player’s data can be tracked using wearable sensors, high-speed cameras, and DL throughout the match. This technology can identify minuscule differences and help us understand the player’s performance under stress. DL can communicate better real-time game plan change to coaches and players and can also identify the minuscule differences.

#### 6.8.3. Refereeing

It is one of the earliest applications of ML and DL in sports. These techniques are helpful in precise judgments to make the game fair and law-abiding. The best example is the use of hawk-eye technology in cricket to determine if the batter is out or not in the cases of LBW.

#### 6.8.4. Health and Fitness Improvement

ML and DL are transforming the healthcare industry in several ways. The predictive and diagnostic capabilities of DL can also be helpful in the sports industry. The physical health and fitness of the players is an extremely important element in sports. Team managements spend a lot of money to maintain the mental and physical wellbeing of the players. Each sport measures the mental and physical skills of a player differently. The adaptation of DL methods in sports can be very helpful to analyze these skills accurately.

#### 6.8.5. Streaming and Broadcasting

AI and DL frameworks are used in match recording, where broadcasters can selectively capture the highlights. DL can automatically generate the subtitles for live sports events in multiple languages based on the viewer’s location and language preferences. DL is also used in sports marketing to determine the best camera angles during the match. In addition, these methods are very helpful to identify the top moments of the game for multiple brands to obtain better advertising opportunities.

## 7. Potential Challenges and Future Research Directions

In the previous sections, we discussed the importance and potential applications of DL techniques in various sectors. However, the successful implementation of these techniques and obtaining desired results in an IIoT is challenging. This section discusses some key issues and future research directions for DL-based IIoT.

### 7.1. Key Challenges

The implementation of DL techniques in the IIoT faces several challenges. In the following, we discuss some key challenges faced by several IIoT applications.

#### 7.1.1. Complexity

This is one of the biggest challenges faced by DL models. Some extra efforts are required to address this problem [150]. The first main issue is the computation requirement and time consumption of the training process because of the complexity of the DL algorithm and large IIoT datasets. The second major problem is the scarcity of large numbers of training data in industrial situations, which can impair the efficiency and accuracy of DL models through overfitting. The complexity problem can be addressed by employing a tensor-train deep compression (TTDC) model for effective feature learning from industrial data [151]. This approach increases model speed by condensing many components in the DL algorithm.

#### 7.1.2. Algorithm Selection

There are several well-known DL algorithms are available for IIoT applications. Most of these algorithms work fine for any general scenario but for a specific IIoT application, the selection of the DL algorithm is a challenge [152]. For implementation in a specific application, it is very important to determine which algorithm is best suitable for this particular application. The inaccurate selection of the DL algorithm can result in several issues, including a waste of money, time, and efforts.

#### 7.1.3. Data Selection

The selection of training data is directly connected to the success of a DL method. In every DL model, the right type and amount of data are important [153]. As part of an industrial process, it is essential to prevent such data that might lead to selective bias.

#### 7.1.4. Data Preprocessing

In DL models, data preprocessing is another mandatory process. It transforms the selected data to make it best compatible with desired DL model [154,155]. This process includes cleaning, data conversion, feature scaling, and removal or replacement of missing entries.

#### 7.1.5. Data Labeling

In terms of implementation, training, and deployment in various IIoT applications, supervised DL techniques are simplest and highly suitable [156]. On the other hand, unsupervised techniques are difficult to implement and sometimes require a lengthy training process. Additionally, data labeling is challenging for both supervised and unsupervised DL models and cannot be outsourced for intensive tasks. DL algorithms are continuously developing, and their feature learning capabilities are changing. The incorporation of new features in the DL framework can be sometimes a nightmare if the earlier datasets, features, and models are not properly documented.

### 7.2. Future Directions

There are multiple aspects of DL frameworks in the IIoT environments that should be rectified for future implementations. This feature will necessitate various improvements, such as the selection of intelligent algorithms with increased efficiency and compatibility with different hardware platforms. In the following, we discuss some potential future research directions in the context of DL-based IIoT.

#### 7.2.1. DL-Enabled Edge/Cloud Computing

In smart industrial environments, fast and efficient computing is considered to be the main feature that affects reliability, latency, and many other important performance parameters [157,158,159]. IIoT applications require powerful machines to compute the large amounts of data generated by diverse operations. To conduct effective computation and provide understandable computing infrastructure for IIoT, a hybrid cloud-edge computing environment is now required. However, this is still not recognized as a realistic method of coping with complex learning issues. There are several reasons behind this problem, such as low processing power, the resource-constrained nature of IoT devices, and the complexity of DL algorithms [160,161]. Therefore, it is deemed appropriate to employ edge-based computing frameworks, simply because of their potential to minimize latency and improve the learning process. However, the integration of DL techniques with an edge-enabled framework for IIoT scenarios remains an open research problem. To achieve self-organization, greater productivity, and reduced runtime, the combined implementation of parallel and distributed learning for edge-based designs needs further optimization [162].

#### 7.2.2. Distributed Deep Learning

Distributed DL is a specialized approach for large-scale DL operations with massive training datasets and long training durations. It functions by assigning several computational resources to collaborate on a single task [163]. Distributed DL can perform several tasks, such as data collection, data mining, and testing, via multiple distributed nodes that work on it and address the initial problem simultaneously and promptly. Therefore, distributed DL is considered one of the most appropriate techniques for implementation in the IIoT. However, its implementation in the smart industrial environment is still a challenging task. The main issue is to determine an effective way of managing the overall distributed computation resources.

#### 7.2.3. Low Latency and Improved Reliability

Smart industrial frameworks require multiple synchronized processes that require lower latency and higher reliability to obtain the desired performance [164,165]. Furthermore, the DL techniques used in IIoT should be capable of dealing with these challenges as well as other aspects such as resource management and network deployment [166]. However, the competency of DL-based IIoT scenarios is still in the early stages with low latency and high-reliability requirements in IIoT. Therefore, research efforts in this area are necessary to build a theoretical and practical foundation for DL-based IIoT to provide low-latency and ultra-reliable communication.

#### 7.2.4. Intelligent Sensing and Decision-Making

Control challenges in DL-based IIoT include both sensing and assessment methods that involve a huge number of sensors and actuators. This does enables smart sensing capabilities though, such as classification, prediction, decision-making, and direct management of the complete IIoT system [167]. Intelligent sensing and useful decision-making abilities are strictly enforced in a smart manufacturing environment, with no room for economic loss or safety issues caused by failure. Therefore, the successful deployment of DL-based IIoT has stringent criteria for accurate prediction, categorization, and decision-making, which can only be met using intelligent sensing and data-driven decisions [168,169].

#### 7.2.5. Lightweight Learning Frameworks

The IIoT framework contains multiple intelligent and connected devices to establish a smart industrial setup [170]. These devices include sensors, actuators, and controllers that use different DL algorithms for the learning process. These devices are considered resource-constrained devices that require lightweight learning algorithms [171]. This will enhance the learning process for diverse industrial devices, resulting in an intelligent IIoT network with reduced computing complexity and increased network lifetime. The best example is the employment of a hardware-in-the-loop (HIL) simulation platform [172,173,174,175,176]. The HIL combines computations for numerous processes with the internal hardware of sensors and actuators. The essential capability of the HIL platform is the use of real-time data generated by installed hardware testbeds.

## 8. Conclusions

This paper presented a comprehensive survey of deep learning (DL) techniques and applications for the Industrial Internet of Things (IIoT). A brief introduction is presented in the context of DL deployments for industrial applications along with the latest state-of-the-art contributions from survey articles. The survey highlighted the major drawbacks of existing studies and overcame these shortcomings by including additional information. Most of the existing surveys lack a detailed description of standard IIoT architecture. This study described the detailed seven-layer architecture of the IIoT along with key enabling technology and protocols. This survey discussed the theories of well-known DL algorithms along with mathematical backgrounds and reference architectures. One of the major shortcomings of the existing studies is the non-consideration of software and hardware implementation platform. To address this issue, a detailed description of software and hardware deployment frameworks is presented in the context of DL and IIoT. To evaluate the effectiveness of DL for IIoT, several potential use cases of DL technologies for the IIoT are discussed. Finally, this survey is concluded by highlighting the key challenges in existing DL-based IIoT systems and presented potential research directions for future endeavors. 

## Figures and Tables

**Table 1:** A comparison of latest survey articles.

Authors	Year	Contributions of Survey Articles					
		IIoT Architecture	Algorithms	Frameworks	Hardware	Applications	Future Directions
Mohammadi et al. [10]	2018	√	√	√	×	√	√
Ma et al. [11]	2019	×	√	×	×	√	√
Sengupta et al. [12]	2020	×	√	×	×	√	√
Ambika et al. [13]	2020	√	√	×	×	√	×
Saleem et al. [14]	2020	×	√	×	×	√	×
Deepan et al. [15]	2021	×	√	√	×	√	×
Khalil et al. [6]	2021	√	√	×	×	√	√
Our Study	2021	√	√	√	√	√	√

**Table 2:** State-of-the-art contributions in DL-based agriculture.

Author	DL Algorithm	Dataset	Application	Purpose of DL Technique
Sehgal et al. [79]	LSTM	Dataset from Syngeta crop challenge (2016)	Weather prediction	Weather prediction according to the conditions of preceding year
Song et al. [80]	DBN	Data gathered from corn field (irrigated) in China	Soil moisture content prediction	Prediction of moisture content in the soil
Douarre et al. [81]	CNN	X-ray tomographic images of soil	Root and soil segmentation	Image categorization into two classes root and soil
Aliev et al. [82]	RNN	Sensory data	Internet of plants-based system	To envisage the minimum and temperature records for ten days
Huang et al. [83]	CNN	Data collected using multirotor UAV	Weed mapping in smart agriculture	Classification of input images into three categories: weed, rice and others
Rahnemoonfar et al. [84]	CNN	Dataset consisting of 24,000 images	Tomato counting	Prediction of tomatoes quantity
Jiang et al. [85]	LSTM	Data obtained from the National Agricultural Statistics Service (NASS) Quick Stats	Crop yield prediction	Corn yield prediction
Ferentinos et al. [86]	CNN	Leaf images of plants	Plant disease detection	Image classification into health and diseased categories
Toda et al. [87]	CNN	Plant Village dataset	Plant disease diagnosis	Leaf image classification into healthy and diseased categories and diagnosis of disease type
Grinblat et al. [88]	CNN	Dataset consisting of vein leaf images of soybean, red beans, and white beans	Plant identification	Legume’s classification into three categories: red beans, soybean, and white beans

**Table 3:** State-of-the-art contributions in DL-based educational sector.

Author	DL Algorithm	Dataset	Application	Purpose of DL Technique
Bhardwaj et al. [89]	CNN	FER-2013, MES dataset	Student engagement	Monitoring the student’s emotions in real time such as anger, fear, disgust, sadness, happiness, and surprise
Han et al. [90]	DNN	Amazon	Smart education platform	Designing an intelligent educational environment
Tsai et al. [91]	MLP	University’s institutional research database	Precision education	To help universities to more precisely understand student backgrounds
Fok et al. [92]	CNN	Self-generated dataset	Prediction model for students’ future development	Analyzing students’ performance and prediction of their future program of studies
Nandal et al. [93]	DNN	Self-generated dataset	Student admission predictor	Development of student admission predictor program for students to find the chances of gaining admission to a university
Khaleel et al. [94]	DCNN	Self-generated dataset	Automated grading	Automatic grade prediction system for the students of computer-aided drawing

**Table 4:** State-of-the-art contributions in DL-based smart healthcare.

Author	DL Algorithm	Dataset	Application	Purpose of DL Technique
Choi et al. [95]	AE + GAN	Sutter PAMF, IMIC-III, Sutter Heart Failure	Generating patient records	Patient’s, record synthesis
Nie et al. [96]	GAN	Brain data from ADNI dataset, Pelvic dataset	Medical image synthesis	Synthetization of CT image from MRI
Sha et al. [97]	RNN	Medical Information Mart for Intensive Care (MIMIC) dataset	Clinical outcome prediction	Mortality prediction
Verma et al. [98]	LSTM	MIT-BIH dataset	Missing data prediction in healthcare	Prediction of missing data in healthcare scenarios
Sun et al. [99]	RBM	Chronic kidney disease (CKD) and dermatology datasets	Clinical decision and risk prediction	Capturing high-level features from the clinical data and predict missing values
Najdi et al. [100]	AE	ISRUC-Sleep dataset	Sleep stage classification	Dimensionality reduction, feature extraction, and classification
Nguyen et al. [101]	RNN	Alzheimer’s Disease Neuroimaging Initiative (ADNI) dataset	Alzheimer’s disease recognition	Modeling the succession of Alzheimer’s disease for seven years
Xue et al. [102]	RNN	Electronic medical records, Sensory data from wearables	Obesity status prediction	Prediction of improvement in obesity status based on blood demographics, pressure, and step count
Amin et al. [103]	CNN	Temple University Hospital dataset	Pathology detection and monitoring	Classification of EEG signals into two categories, normal and pathological
Wang et al. [104]	LSTM	Normal Sinus Rhythm (NSR), Fantasia Database (FD),	Congestive heart failure	Detection of congestive heart failure
Alhussein et al. [105]	CNN	SVD database, MEEI database	Voice pathology detection	Classification of voice signals into normal and pathological categories
Maragatham et al. [106]	LSTM	Electronic Health Records	Heart failure prediction	Modeling the risk prediction of heart failure
Kim et al. [107]	DBN	Sixth Korea National Health and Nutrition Examination Survey (KNHANES-VI) 2013 dataset	Cardiovascular risk prediction	Development of cardiovascular risk prediction model

**Table 5:** State-of-the-art contributions in DL-based intelligent transportation system.

Author	DL Algorithm	Dataset	Application	Purpose of DL Technique
Su et al. [108]	LSTM	38.6 h of transportation data	Mode detection system	Identification of mode of transport based on kinetic energy harvester
Song et al. [109]	LSTM	GPS data and transportation network data	Human mobility and transportation mode prediction	Prediction of human movements
Mohammadi et al. [110]	GAN	Localization dataset, Path planning dataset	Path planning	Safe and reliable paths generation
Camero et al. [111]	RNN	Data from 29 Parking slots in Birmingham	Car Park occupancy prediction	Prediction of occupancies rate of car parks
Singh et al. [112]	AE	Traffic videos	Road Accident detection	Extraction of Spatio-temporal features from the surveillance video
Lv et al. [113]	RNN + CNN	Trajectory data from Beijing and Shanghai	Traffic speed prediction	Traffic speed prediction
Ma et al. [114]	RBM + RNN	GPS data	Congestion Evolution Prediction in the transportation network	Traffic congestion evolution from GPS data
Pérez et al. [115]	RBM	Floating car data gathered in Barcelona	Real-time traffic forecasting	Traffic prediction in real time
Xiangxue et al. [116]	LSTM	Floating Car Data	Short-term traffic prediction	Modeling of traffic flow in urban road networks
Goudarzi et al. [117]	DBN	Data containing historical road traffic flow	Traffic flow prediction	Traffic flows prediction

**Table 6:** State-of-the-art contributions in DL-based manufacturing industries.

Author	DL Algorithm	Dataset	Application	Purpose of DL Technique
Park et al. [118]	AE	Sensory data, network traffic data	Intrusion detection system	Development of IDS
Tao et al. [119]	CNN	Sensory data	Worker activity recognition	Classification of worker’s activities into 6 groups: screwdriver, used power, grab tool, hammer, rest arm, turn a screwdriver, and wrench usage
Ren et al. [120]	AE	IEEE PHM2012 data provided by the FEMTO-ST Institute in France	Remaining useful life prediction of bearings	Features extraction that is important for the remaining bearings’ life prediction
Yan et al. [121]	AE	Data collected from CNC machining centers	Remaining useful life prediction in machines	Features extraction that is important for the remaining machine’s life prediction
Jiang et al. [122]	AE	Process data samples	Fault classification	Feature learning from a wide variety of faults
Yuan et al. [123]	CNN	Bearing data offered by Case Western Reserve University (CWRU)	Diagnosis and monitoring in manufacturing	Identification and prediction of machine faults
Li et al. [124]	CNN	Sensory data	Manufacture inspection system	Classification of production items into two categories: defected and non-defected.
Wang et al. [125]	DBN	Sensory data gathered from a centrifugal compressor	Condition prediction	Prediction of machine’s condition in manufacturing systems
Zhang et al. [126]	LSTM	Sensory data obtained from 33 sensors deployed on a pump in power station	Industrial IoT equipment analysis	Prediction of the working condition of industrial equipment to enhance operation quality

**Table 7:** State-of-the-art contributions in DL-based aviation industries.

Author	DL Algorithm	Dataset	Application	Purpose of DL Technique
Alkhamisi et al. [127]	RNN	Aviation Safety Reporting System (ASRS) dataset	Risk prediction in Aviation Systems	Improvements of risks prediction in aviation systems
Rodrigo et al. [128]	PCMC-Net	Data extracted from a global distribution system (GDS)	Price elasticity estimation	Differentiate the price elasticity between business and leisure trips
Barakat et al. [129]	CNN + LSTM	Twitter US Airline Sentiment dataset	Airport service quality	Measurement of airport service quality using passengers’ tweets about airports
Wu et al. [130]	CSAE	Self-generated EEG dataset	Detecting fatigue status of pilots	Development of fatigue recognition system based on EEG signals and DL algorithms
Dong et al. [131]	LSTM	Aviation Safety Reporting System (ASRS)	Aviation transportation safety	Identification of incident causal factors for aviation transportation safety improvement.
Yazdi et al. [132]	SAE-LM + SDA	U.S flight dataset	Flight delay prediction	Development of flight delays prediction system.
Wang et al. [133]	LSTM	ASPM datasets	Flight demand and delays forecasting	Prediction of flight departure demand in a multiple-stage time horizon
Corrado et al. [134]	DAE	Flight data collected from San Francisco International Airport	Anomaly detection	Development of an anomaly detection system to identify deviated trajectories
Hasib et al. [135]	DNN + CNN	US airline service dataset	Sentiment analysis	Evaluation of six major US airlines and multi-class sentiment analysis

**Table 8:** State-of-the-art contributions in DL-based defense systems.

Author	DL Algorithm	Dataset	Application	Purpose of DL Technique
Das et al. [136]	R-CNN	Self-generated dataset	Target detection	Development of a new search algorithm for object detection through UAV
Calderón et al. [137]	CNN	Self-generated dataset	Real-time object detection	Development of a vision-based object detection system for a micro-UAV
Krishnaveni et al. [138]	DCNN	Data collected from wildlife television.	Surveillance applications	Identification of abnormal events and the data streaming by creating a multipath routing in WSN
Pradeep et al. [139]	CNN	Self-generated dataset	Real-time object recognition in air defense systems	Accurate identification of definite target with DL algorithm and real-time camera of FWN aircraft
Shi et al. [140]	FNN	Self-generated	Cognitive radio security	Launching of jamming attacks on wireless communications and development of a defense strategy
Wang et al. [141]	DRL	Self-generated dataset	Defense strategies against adversarial jamming attacks	Design and development of defense strategies against DRL-based jamming attackers on a multichannel access agent

**Table 9:** State-of-the-art contributions in DL-based sports industry.

Author	DL Algorithm	Dataset	Application	Purpose of DL Technique
Chen et al. [142]	GAN	NBA SportVu	Basketball	Development of realistic defensive plays conditioned on the ball and offensive term movements
Chung et al. [143]	GAN	STATS SportVu	Basketball	Simulation of offensive tactic sketched by coaches
Baccouche et al. [144]	LSTM + RNN	MICC-Soccer-Actions-4 dataset	Football	Classifying four football actions
Theagarajan et al. [145]	CNN	3 different soccer matches	Football	Generation of sports highlights
Le et al. [146]	RNN	STATS	Football	Ghost modeling in football.
Kautz et al. [147]	DCNN	Video Recordings from GoPro Hero 3 action camera	Volleyball	Activity recognition in volleyball
Qiao et al. [148]	DCNN + LSTM	Self-built video dataset	Table Tennis	Recognition and tracking of table tennis’s real-time trajectories in complex environments
Cao et al. [149]	Tiny YOLOv2	Self-generated shuttlecock detection dataset	Badminton	Precise and detection of the shuttlecock with badminton robot

## Data Availability

Not applicable.
